# Using UPLC-LTQ-Orbitrap-MS and HPLC-CAD to Identify Impurities in Cycloastragenol, Which Is a Pre-Clinical Candidate for COPD

**DOI:** 10.3390/molecules28176382

**Published:** 2023-08-31

**Authors:** Feng Zhu, Xiao Zhang, Bing-Yuan Du, Xiang-Xia Zhu, Gui-Fang Zhao, Ying Sun, Qing-Qiang Yao, Hong-Bao Liang, Jing-Chun Yao, Zhong Liu, Gui-Min Zhang, Guo-Fei Qin

**Affiliations:** 1 State Key Laboratory of Integration and Innovation of Classic Formula and Modern Chinese Medicine, Lunan Pharmaceutical Group Co., Ltd., Linyi 273400, China; zhufeng0716@163.com (F.Z.); szyzhangxiao@163.com (X.Z.); dubingyuanyuan@126.com (B.-Y.D.); zhuxiangxia1986@163.com (X.-X.Z.); zhaoguifang2000@163.com (G.-F.Z.); sunying1982@126.com (Y.S.); lianghongbao1985@163.com (H.-B.L.); yaojingchun@lunan.cn (J.-C.Y.); clevertree@163.com (Z.L.); 2 College of Pharmacy, Shandong University of Traditional Chinese Medicine, Jinan 250355, China; 3 Jining Medical University, Jining 272067, China; yao_imm@163.com

**Keywords:** 10,19-secocycloartane triterpenoids, impurities, cycloastragenol, astragaloside IV, Smith degradation, HPLC-CAD, UPLC-LTQ-Orbitrap-MS

## Abstract

Chronic obstructive pulmonary disease (COPD) is a highly prevalent disease that has become the third leading cause of death worldwide. Cycloastragenol (CAG), which is the genuine sapogenin of the main active triterpene saponins in *Astragali radix*, is a bioavailable pre-clinical candidate for chronic obstructive pulmonary disease (COPD), and it was investigated in our previous study. In order to progress medical research, it was first efficiently produced on a 2.5-kg scale via Smith degradation from astragaloside IV (AS-IV). Simultaneously, since the impurity profiling of a drug is critical for performing CMC documentation in pre-clinical development, a study on impurities was carried out. As these structures do not contain chromophores and possess weak UV absorption characteristics, HPLC-CAD and UPLC-LTQ-Orbitrap-MS were employed to carry out the quality control of the impurities. Then, column chromatography (CC), preparative thin-layer chromatography (PTLC), and crystallization led to the identification of 15 impurities from CAG API. Among these impurities, compounds **1, 4**, **9**, **10**, **14**, and **15** were elucidated via spectroscopic analysis, and **2**–**3, 5**–**8**, and **11**–**13** were putatively identified. Interestingly, the new compounds **9** and **14** were rare 10, 19-secocycloartane triterpenoids that displayed certain anti-inflammatory activities against LPS-induced lymphocyte cells and CSE-induced MLE-12 cells. Additionally, a plausible structural transformation pathway of the degradation compounds from CAG or AS IV was proposed. The information obtained will provide a material basis to carry out the quality control and clinical safety assurance of API and related prescriptions. Reasonable guidance will also be provided regarding the compounds with weak UV absorption characteristics.

## 1. Introduction

Chronic obstructive pulmonary disease (COPD) is a common respiratory disease affecting more than 10% of people aged 40 years old and above worldwide [1,2]. Every year, 3 million people are killed by this disease. It is currently the third leading cause of death, and it will be the seventh greatest burden disease in 2030 [2]. According to pertinent data, the main drugs used to treat COPD are bronchodilators, anticholinergics, and hormones, but COPD-related mortality is still increasing despite improvements in medicine [3]. Therefore, it is imperative to discover and design new drugs.

**Figure 1:**
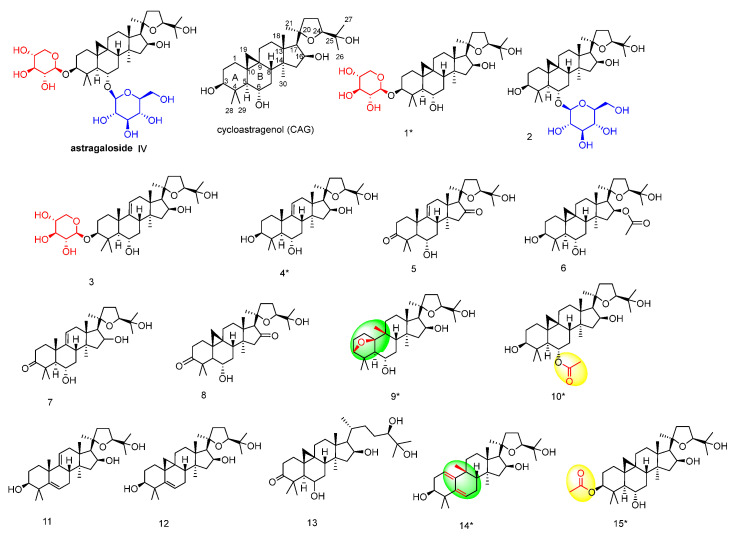
Structures of astragaloside IV, cycloastragenol, and compounds **1**–**15**. * The structure of the main isolated impurities (≥0.05%) derived from Fraction B.

Astragali radix (AR), i.e., the dried roots of *Astragalus membranaceus* (Fisch.) Bge. Var. mongholicus (Bge.) Hsiao or *A. membranaceus* (Fisch.) Bge., is one of the most well-known traditional Chinese medicines (TCMs), and it has been utilized to treat disease or as a food additive for more than 2000 years [4]. The results obtained in clinical practice have shown that it induces diverse therapeutic effects, including immunomodulatory, anti-tumor, anti-hyperglycemic, anti-inflammatory, and anti-viral actions [5,6]. Remarkably, AR and its extracts are also widely and legitimately used in the development of health products due to their myriad biological functions, such as delaying senescence and providing fatigue resistance [5,7]. Previous chemical investigations of these genera revealed that flavonoids, polysaccharides, and saponins are their bioactive metabolites [8]. Among these metabolites, astragaloside IV (AS-IV) is the main active ingredient of Astragali radix, and its use as a qualitative control has been documented in *the Chinese Pharmacopoeia* [9,10]. Cycloastragenol (CAG) (Figure 1), which is a cycloartane triterpenoid, is the genuine sapogenin of the main active triterpene saponins, possessing anti-aging, anti-inflammatory, anti-oxidative, and airway inflammation-alleviative pharmacological effects [11,12].

In our previous study, we demonstrated that CAG exhibited preventive and therapeutic effects on chronic obstructive pulmonary disease (COPD) [13]. Additionally, it can be obtained using Smith degradation to hydrolyze the xylose at C-3 and the glucose at C-6 in AS-IV [14]. Motivated by the above findings, CAG was first efficiently prepared on a 2.5-kg scale to perform further research. Though the total amount of impurities was controlled in CAG, its composition was still unclear.

According to regulatory guidelines Q3A and Q3B of the International Council for Harmonisation of Technical Requirements for Pharmaceuticals for Human Use (ICH), impurities in chemical drugs of at least than 0.05% should be assured in order to guarantee the safety and quality of active pharmaceutical ingredients (APIs) and related preparations [15,16]. In this study, quantitative and qualitative methods based on high performance liquid chromatography tandem using a charged aerosol detector (HPLC-CAD) and ultra performance liquid chromatography–linear trap quadrupole–orbitrap mass spectroscopy (UPLC-LTQ-Orbitrap-MS) were used to control the impurities [17]. Due to the absence of reference material, the proportion of related compounds was evaluated via the normalization method of peak area. Eventually, column chromatography (CC), preparative thin-layer chromatography (PTLC), crystallization, UPLC-MS analysis, and comparisons with reference material led to the isolation and identification of two new rare 10,19-secocycloartane triterpenoids (compounds **9** and **14**), together with 13 known degradation products of AS-IV (Figure 1). Considering the importance of CAG and its derivatives available on the market, two new compounds, i.e., compounds **9** and **14**, were further explored to determine their anti-inflammatory activities against LPS-induced lymphocyte cells and CSE-induced MLE-12 cells, for which positive results were obtained.

## 2. Results and Discussion

### 2.1. Large-Scale Preparation of Cycloastragenol

To prepare CAG from AS-IV, most of the methods used to cleave glycosides, like acid hydrolysis (using HCl, acetic acid, etc.), acetolysis (using acetic anhydride/ZnCl_2_), enzymatic hydrolysis (using glusulase, hesperidinase, etc.) and Smith degradation, were studied. Consequently, it was observed that Smith degradation could efficiently produce CAG with very few secocycloartane byproducts, and this result was consistent with those mentioned in the literature [14]. Then, after optimizing the complicated post-processing steps (especially extraction and column chromatography), the large-scale preparation of CAG on a 2.54-kg scale was achieved.

### 2.2. Structural Elucidation of the Main Isolated Impurities (≥0.05%) Derived from Fraction B

**Figure 2:**
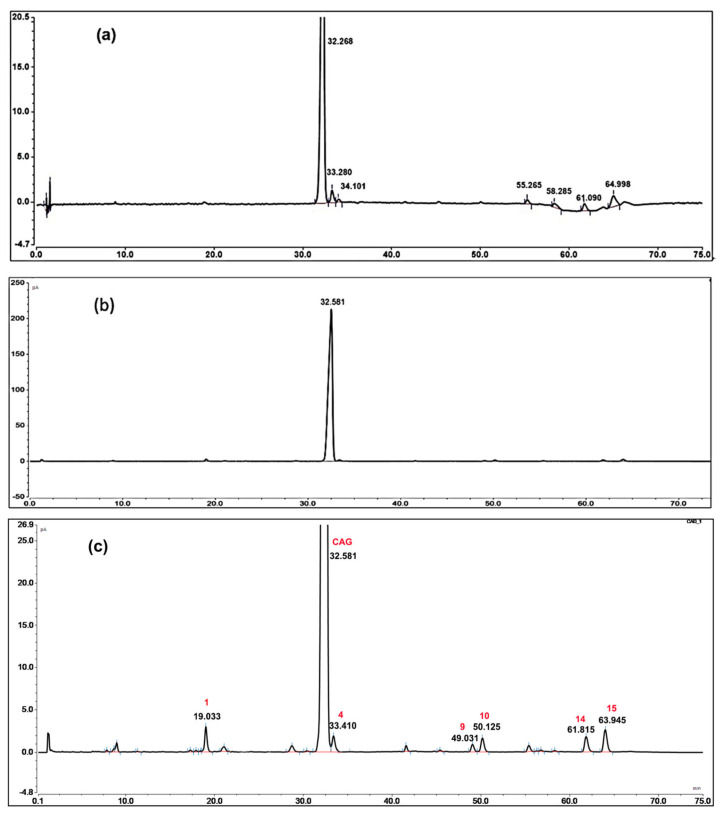
(**a**) Amplificatory HPLC-DAD chromatogram of CAG and the main isolated impurities (≥0.05%); (**b**) original HPLC-CAD chromatogram of CAG and the main isolated impurities (≥0.05%); (**c**) amplificatory HPLC-CAD chromatogram of CAG and the main isolated impurities (≥0.05%).

Column chromatography (CC), preparative thin-layer chromatography (PTLC), crystallization, and comparison to reference material led to the isolation and identification of two new rare 10,19-secocycloartane triterpenoids (compounds **9** and **14**), together with four known degradation products of AS-IV (compounds **1**, **4**, **10,** and **15**). In the quality control of CAG, the high-performance liquid chromatography–diode array detector (HPLC-DAD) method was used. Unfortunately, as CAG and compounds **1**–**15** did not contain chromophores and possessed weak UV absorption characteristics, this method could not be used to obtain a good response from these factors (Figure 2a). Thus, an HPLC-CAD-based method, the performance of which did not depend on a subject’s structure [17], was developed to carry out the quality control of degradation impurities. As shown in Figure 2b,c, the impurities could be effectively baseline separated.

**Figure 3:**
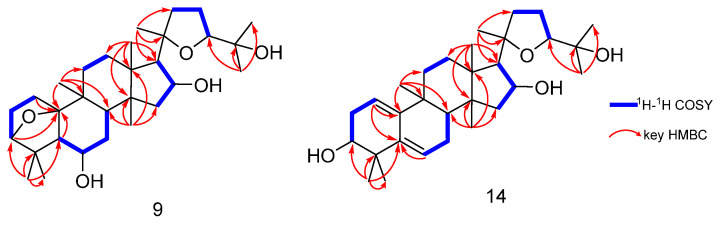
1H-1H COSY and selected key HMBC of compounds **9** and **14**.

Compound **9**, which was a white amorphous solid, was an isomer of CAG with the same molecular formula, i.e., C_30_H_50_O_5_, which was inferred from the HRESIMS data [M+Na]^+^ at *m*/*z* 513.3557 (calculated as 513.3550). The absence of characteristic AX coupling system signals (*δ*_H_ 0.39 (1H, d, *J* = 4.1 Hz) and 0.67 (1H, d, *J* = 4.1 Hz)) (Table 1) and quaternary carbons in the high-field region (*δ*_C_ 21.0 (C) and 29.9 (C)) [18] implied the cleavage of the cyclopropane. Then, key heteronuclear multiple-bond correlations (HMBC) from H_3_-19 to C-8, C-9, C-10, and C-11 indicated that the cleavage position of the cyclopropane was between C-10 and C-19, which was consistent with the absence of HMBC between H_3_-19 and C-5 (Figure 3). Additionally, it was observed that C-10 was oxygenated, according to the chemical shifts of C-10 (*δ*_C_ 93.2). Further analysis of the key long-range HMBC correlation between H-3 and C-10 revealed that the structure of **9** contained a (C-3)–O–(C-10) oxygen bridge moiety, which may be formed via dehydration between 3-OH and 10-OH in acidic conditions. Thus, compound **9** was found to be a rare 10, 19-secocycloartane triterpenoid with an ether bridged bond, as shown in Figure 1.

Compound **14** was obtained as a white amorphous solid. It was determined that its molecular formula was C_30_H_48_O_4_ due to its high-resolution electrospray ionization mass spectrometry (HRESIMS) peak at *m*/*z* 473.3621 [M+H]^+^ (calculated as 473.3625), indicating seven degrees of unsaturation. The ^1^H NMR and ^13^C NMR spectra (Table 2) revealed the presence of two trisubstituted double bonds (*δ*_H_ 5.59 (1H, brs) and 5.59 (1H, brs) as well as *δ*_C_ of 142.3 (C), 139.7 (C), 119.6 (CH), and 115.2 (CH)), accounting for two degrees of unsaturation. Compared to the ^13^C NMR data regarding CAG (Table S1), the major differences were signals corresponding to ring A and ring B carbons, as well as the absence of quaternary carbons in the high-field region, of which the latter element implied that compound **14** was a pentaclic secocycloartane triterpenoid. The HMBC correlations from H_3_-19 to C-8, C-9, C-10, and C-11 (Table 1) confirmed the C10/C19 cleavage position of the cyclopropane. Then, the ^1^H-^1^H correlation spectroscopy (^1^H-^1^H COSY) correlations, namely H-1/H_2_-2/H-3 and H-5/H_2_-6/H-7 (Figure 3), together with the key HMBC correlations from H-1 to C-5 and C-10 and H_3_-28 and H-6 to C-5, revealed a C1/C10/C5/C6-conjugated diene moiety. Thus, it was established that compound **14** was a rare 10, 19-secocycloartane triterpenoid, as shown in Figure 1.

The known compounds **1**, **10,** and **15** were identified as 3-*O*-*β*-D-xylopyranosyl-cycloastragenol, (20*R*,24*S*)-6*α*,16*β*,25-trihydroxy-3*β*-acetoxy-20,24-epoxy-cycloartane, and (20*R*,24*S*)-3*β*,16*β*,25-trihydroxy-6*α*-acetoxy-20,24-epoxy-cycloartane, respectively, via a comparison of their spectroscopic data to those reported in the literature [19,20,21]. Compound **4** was identified as astragenol via comparison to a reference material.

**Scheme 1:**
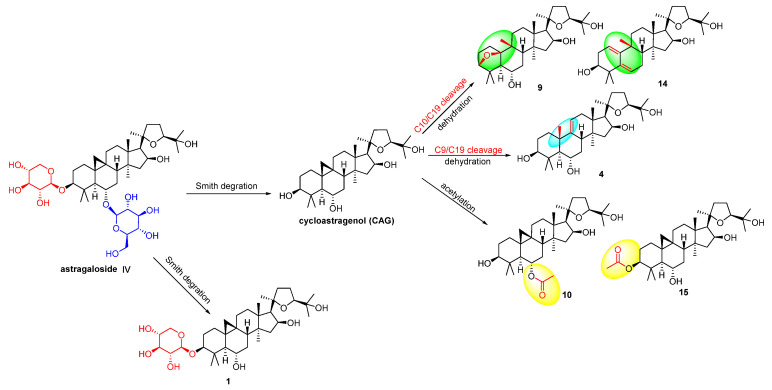
Plausible structural transformation pathway of the major isolated impurities (≥0.05%) of CAG or AS IV.

Additionally, a plausible structural transformation pathway of the isolated impurities from CAG or AS-IV was proposed. As shown in Scheme 1, compounds **4**, **9**–**10,** and **14**–**15** originated from CAG. The conversion of CAG into compounds **9** and **14** could be achieved via the cleavage of the cyclopropane ring at C10/C19, which subsequently underwent dehydration. However, if the cleavage position was located between C9 and C19, compound **6** would be generated. Furthermore, compounds **10** and **15** were acetylation products of CAG. As there are two glycosyl groups in AS-IV, if only the glucose at C-6 was detached via hydrolysis, compound **1** would be produced.

### 2.3. UPLC-LTQ-Orbitrap-MS Conditions Required for the Determination of Other Related Compounds (<0.05%)

**Figure 4:**
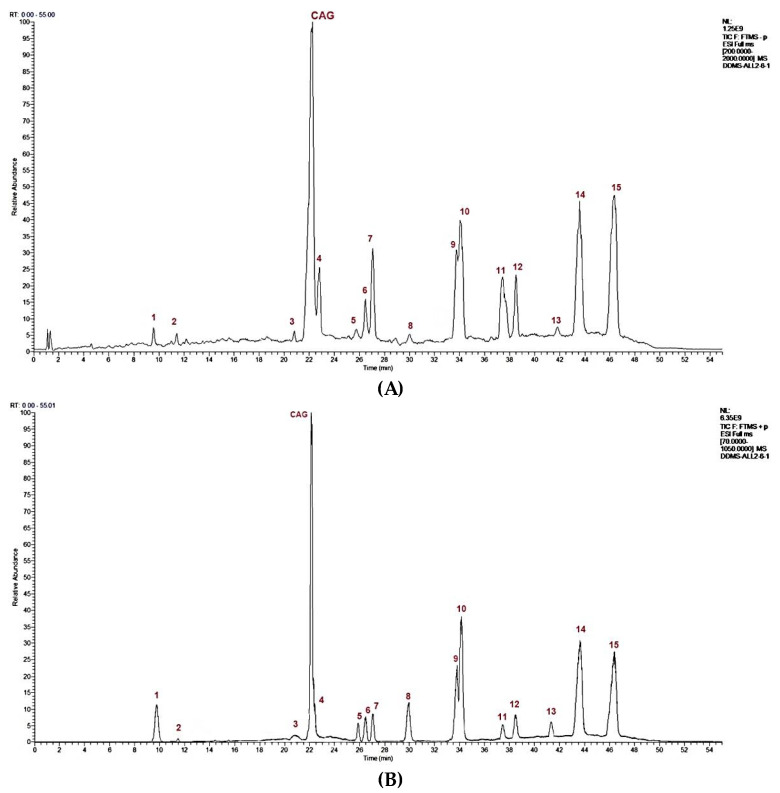
UPLC-LTQ-Orbitrap-MS negative ion (**A**) and positive ion (**B**) mass spectrum base compound ion flow results.

According to the MS/MS fragment behavior of Astragaloside IV and Cycloastragaloside and considering the plausible structural transformation pathway of the isolated impurities, the other related compounds (<0.05%) were putatively identified. As shown in Figure 4, the UPLC-LTQ-Orbitrap-MS results revealed the existence of 15 chemical compounds. For the known compounds, since there are already a number of excellent papers in which they have been discussed [22,23,24,25], we do not provide a detailed description of them in this study. These structures, compared to the standards directly isolated or deduced according to the fragmentation pathway, were unambiguous or tentatively identified along with their CAS and molecular weight, the *m*/*z* of the molecular ions, and the retention time of each compound, all of which are shown in Table 3. MS/MS^2^ mass spectrometry data are shown in the Supporting Information (Figures S24–S38) document. Our UPLC-LTQ-Orbitrap-MS results showed accurate mass error values, which were below 5 ppm.

Generally, cycloartane triterpenoids with skeletons similar to astragaloside IV and cycloastragaloside display characteristic ions, and the fragment ions of each compound are also essentially the same as the cleaved pathway reported in the published papers. In the positive mode, cycloartane triterpenoids generate aglycone residues at *m*/*z* 473 (C_30_H_49_O_4_), 455 (C_30_H_47_O_3_), 437 (C_30_H_45_O_2_), and 419 (C_30_H_43_O), as well as 25-hydroxy-20, 24-epoxy residues at *m*/*z* 143 (C_8_H_15_O_2_) and 125 (C_8_H_13_O) [25], while the fragment ion data from the positive ion mode can be used to judge the existence of cyclopentane triterpenoids. It was noticed that [M+H]^+^ and [M+Na]^+^ ions were easily weakened or lost. In contrast, the compounds exhibited a high response in negative-ion mode in the form of [M+HCOOH-H]^−^ and [M-H]^−^ due to the presence of formic acid in the mobile phase and a weak deprotonated ion [M-H]^−^ [26]; these characteristics can be used to aid the identification of the compounds.

Specifically, compounds **1**–**3** were generated from AS-IV, while **4**–**15** were transformed from CAG. Since there are two glycosyls in AS-IV, compound **1** can be obtained if the glucose is hydrolyzed at C-6, and **2** can be obtained if the xylose is hydrolyzed at C-3. Due to the unstable 9, 19-cyclopropane ring, an acid–base reaction may result in the cleavage of the cyclopropane ring at C10/C19 or C9/C19, where, theoretically, the cleavage position is random; subsequently, the cleaved ring undergoes dehydration to form a double bond. Furthermore, due to the oxidization property of NaIO_4_, the hydroxy group at C-3/C-16 could be oxidized to a carbonyl group (compounds **5**, **7**, **8**, and **13**), and the 20, 24-epoxy ring could readily open (compound **13**). Of course, compound **13** showed ions of [M+Na]^+^ (*m*/*z* 513.3557) and [M+HCOOH-H]^−^ (*m*/*z* 535.3643) in its full-scan mass spectra, respectively. Furthermore, the reductions in the *m*/*z* equal to 143 and 125 prove our supposition [25,26]. In addition, if C-3/C-6/C-16 undergo acetylation, the structures of **6**, **10**, and **15** will be established.

Moreover, Smith degradation showed high selectivity and produced CAG as a predominant product, but these results were accompanied by the cleavage of the cyclopropane ring, dehydration, acetylation, and partial oxidation that may not individually occur. In fact, under specific reaction conditions, CAG undergoes chemical transformations, such as acetylation, glycosylation, ring opening, oxidation, and halogenation [27]. The results discussed herein present a real profile of the various transformations induced via the Smith degradation of astragaloside IV, and this profile is important for carrying out the quality control of CAG. Moreover, it is worth mentioning that CAG can be converted from AS-IV using intestinal bacteria, which is a process that also involves the stepwise degradation of two glycosyls [28].

### 2.4. Anti-Inflammatory Activity of Two New Impurities

Considering the importance of CAG and its derivatives on the market, two new compounds, i.e., compounds **9** and **14**, were further explored in relation to their anti-inflammatory activities against LPS-induced lymphocyte cells and CSE-induced MLE-12 cells. As we expected, both compounds showed good anti-inflammatory activities against LPS-induced lymphocyte cells, with inhibition rates of 50.40% and 49.61% in the 30-micrometer range and 89.15% and 89.57% in CSE-induced MLE-12 cells in the 1-micrometer range, respectively (Tables S2–S4). These observations are important for determining the druggability and safety of CAG.

## 3. Materials and Methods

### 3.1. Chemicals and Reagents

Astragaloside IV (AS IV) (purity ≥ 98%) was purchased from Xi’an Hao-Xuan Bio-Tech Co. Ltd., Xi’an, China. Reference materials for AS IV and astragenol were purchased from the National Institutes for Food and Drug Control, Beijing, China. All other chemicals were of analytical reagent grade.

### 3.2. Large-Scale Preparation of Cycloastragenol

In total, 5.0 kg of AS IV (dissolved in 298.5 kg of ethanol) and 658.0 kg of purified water containing 8.1 kg of NaIO_4_ were added to a glass-lined reactor. After stirring for 12 h at room temperature, the reaction was terminated using 16.2 kg of ethylene glycol. Then, the reaction mixture was concentrated to remove the ethanol and centrifuged. The residue, which was washed with 500.0 L of purified water, was dissolved in 127.5 L of stirred ethanol. Under the protection of nitrogen, 2.15 kg of NaBH_4_ was added. The solution was stirred at room temperature for 12 h and terminated with 3.2 L of acetic acid to adjust the pH to 6–8. After 53.0 kg of 7.5% HCl was added, the mixture was stirred at 60.0 ± 1 °C for 4 h, and the pH was adjusted to 6–8 using 14.0 kg of 10.0-molarity NaOH after the reaction. The solvent was removed via evaporation and centrifugation, and the residue was washed with 500.0 L of purified water and dried to provide 2.54 kg of CAG (purity = 98.59%).

### 3.3. Sample Preparation and Enrichment for Impurities

Silica gel ((100–200 mesh, 200–300 mesh, and 300–400 mesh from Qingdao Makall Group Inc., Qingdao, China) was used via column chromatography to enrich impurities, and spots were visualized by heating SiO_2_ plates sprayed with 10% H_2_SO_4_ in EtOH. The CAG (10.0 g) was subjected to silica gel column elution at a gradient of petroleum/acetone of 3.5/1 to 1/1 (*v*/*v*). Two fractions were collected and named fractions A and B. Between the two fractions, fraction A (9.59 g) was CAG, and B (208 mg) was impurity-b. Then, 1 mg of fraction B was dissolved in 10 mL of methanol, and the solution was applied via liquid chromatography–mass spectrometry (LC–MS) analysis. The remaining amount of fraction B was used to purify impurities.

### 3.4. Isolation of the Main Impurities (≥0.05%) Derived from Fraction B

Silica gel (100–200 mesh, 200–300 mesh, and 300–400 mesh from Qingdao Makall Group Inc., Qingdao, China) was used to carry out column chromatography, preparative thin-layer chromatography (PTLC) (HSGF254 (Size: 0.4–0.5 mm), Yantai Jiangyou Silica Gel Development Co., Yantai, China) was used to isolate compounds, and spots were visualized by heating SiO_2_ plates sprayed with 10% H_2_SO_4_ in EtOH. Fraction B (207 mg) was subjected to silica gel column elution at a gradient of petroleum/acetone of 3.5/1 to 1/1 (*v*/*v*), yielding 3 fractions. Fraction 1 was purified via PTLC twelve times, for which a mixture of petroleum/ethyl acetate (4/1) was used as a developing solvent, yielding compound **10** (8.3 mg). Fraction 2 was chromatographed through silica gel elution and dichloromethane/ethyl acetate (3/1, *v*/*v*) to obtain three subfractions (F21–F23). Subfraction F22 was refined via a silica gel column using petroleum/EtOAc (3/1, *v*/*v*) and another silica gel column using petroleum/acetone (3/1, *v*/*v*), resulting in **14** (12.0 mg). Subfraction F23 was purified via a silica gel column using petroleum/acetone (2/1, *v*/*v*) and isolated via PTLC thirteen times, using a mixture of petroleum/acetone (3/1, *v*/*v*) as developing solvent, to identify **9** (5.0 mg) and **15** (8.0 mg). Compound **4** was identified as astragenol via comparison with a standard substance. Fraction 3 was employed via column chromatography elution using etroleum/acetone (2/1, *v*/*v*) and separated via crystallization in a solvent of MeOH to obtain **1** (17.0 mg).

Compound **9**: White amorphous solid; IR (KBr) *ν*_max_ 3386, 2960, 2873, 1467, 1451, 1381, 1362, 1259, 1204, 1171, 1099, 1064, 1035, and 1012 cm^−1^; ^1^H and ^13^C NMR data (see Table 1). HRESIMS: *m*/*z* 513.3557 [M+Na]^+^ (calculated for C_30_H_50_O_5_Na as 513.3550).

Compound **14**: White amorphous solid; IR (KBr) *ν*_max_ 3393, 3330, 2966, 2931, 2869, 1732, 1655, 1466, 1450, 1380, 1262, 1182, 1149, 1099, 1044, and 1033 cm^−1^; ^1^H and ^13^C NMR data (see Table 2). HRESIMS: *m*/*z* 473.3621 [M+H]^+^ (calculated for C_30_H_49_O_4_ as 473.3625).

### 3.5. Analysis of the Isolated Impurities (≥0.05%)

The HPLC-CAD experiments were performed using an UltiMate 3000 instrument (Thermo Scientific Corporation, Madison, WI, USA) with a Corona Veo CAD detector (Thermo Scientific Corporation, Madison, WI, USA) equipped with an analytic reversed-phase column (Waters CORTECS G8, 150 × 4.6 mm, 2.7 μm). The nitrogen used in the CAD detector was provided by a Corona Air Compressor 230V (Peak Scientific Instruments Ltd., Glasgow, Scotland, UK). The HPLC-CAD chromatogram of CAG and its impurities were collected and processed using the Chromeleon 7.10 workstation. The following gradient elution program was employed: a flow rate of 1.0 mL/min, mobile phase A/mobile phase B = acetonitrile/deionized water, 0–5 min (30% A), 5–10 min (30% A–32% A), 10–15 min (32% A), 15–20 min (32% A–35% A), 20–25 min (35% A), 25–30 min (35% A–38% A), 30–35 min (38% A), 35–40 min (38% A–45% A), 40–50 min (45% A), 50–55 min (45% A–50% A), 55–65 min (50% A), 65–70 min (50% A–30% A), and 70–75 min (30% A).

### 3.6. HRESIMS, NMR, UV, and IR Spectra of Isolated Impurities

HRESIMS spectra were measured using a 1290 Infinity II UHPLC/6520 Q-TOF MS (Agilent Technologies Inc., Palo Alto, CA, USA). NMR spectra were captured using Bruker AVANCE III 400 spectrometers (Bruker, Karlsruhe, Germany). A frequency of 400 MHz was employed to perform ^1^H NMR, and a frequency of 100 MHz was employed to perform ^13^C NMR in C_5_D_5_N. Chemical shifts (*δ*) in ppm referred to the solvent peaks at *δ*_H_ 7.20, 7.57, and 8.72; *δ*_C_ 123.44, 135.44, and 149.83 for C_5_D_5_N; and the coupling constant *J* in Hz. UV spectra were recorded using a Thermo Scientific Evolution 350 spectrophotometer (Thermo Scientific Corporation, Madison, WI, USA). IR spectra were measured using a Nicolet IS 10 spectrophotometer (Thermo Nicolet Corporation, Madison, WI, USA) in KBr discs.

### 3.7. UPLC-LTQ-Orbitrap-MS Conditions for Determination of Related Compounds (<0.05%)

Ultra–performance liquid chromatography–linear trap quadrupole–orbitrap mass spectroscopy (UPLC-LTQ-Orbitrap-MS) studies were conducted using a Hypersil GOLD Thermo Scientific HPLC system (Thermo Fisher Scientifc, Waltham, MA, USA) and an LTQ mass spectrometer. Analyses were performed using a Vanquish liquid chromatographer (Thermo Scientific Corporation, Waltham, MA, USA) equipped with a HSS T3 column (Waters, 2.1 mm × 100 mm, 1.7 μm). The mobile phase consisted of 0.01% formic acid–water (A) and acetonitrile (B), and the flow rate was 0.2 mL/min. The sample solutions were eluted using the following gradient program: 85% A at 0–5 min. Then, the linear gradient was increased to 85–80% A at 5–10 min, 80–65% A at 10–20 min, 65% A at 20–30 min, 65–50% A at 30–40 min, 20% A at 40–50 min, and 85% A at 50–55 min at a flow rate of 0.25 μL/min. The column temperature was 40 °C, and the injection volume was 2 μL.

TOF/MS analysis was conducted in positive and negative modes at an ion source temperature of 350 °C and an Ionspray Voltage of 4 kV. The sheath gas (at a flow rate of 40 arb) and auxiliary gas (with a flow rate of 20 arb) were of the highest purity (99.99%), and the mass range was set at *m*/*z* 100–1500. The first-level resolution was 30,000, and MS^2^ was scanned at 17,500. The fragmentation voltages were 20, 40, and 60 eV. Finally, the resultant data were analyzed using the standard Xcalibur 4.2 SP1 software.

### 3.8. Anti-Inflammatory Activity of Two New Impurities

#### 3.8.1. LPS-Induced Lymphocyte Cells [29]

Lymphocyte cells were seeded into a 96-well plate at a density of 5 × 10^6^ cells/mL for 24 h of incubation. Then, they were pre-treated with target compounds upon reaching 3, 10, and 30 μM using lipopolysaccharide (LPS) (5 μg/mL) for another 6 h. Solvent-only cells served as negative controls. In brief, cells were collected and stained using PerCP CY5.5-conjugated anti-mouse CD3 and PE-conjugated anti-mouse CD69 in staining buffer at RT for 20 min in the dark. The CD19+CD69+ lymphocytes were tested using flow cytometry (BECKMAN CytoFLEX). The cell survival rate was calculated according to the following formula:

cell survival rate (%) = [A_450_ (sample)/A_450_ (Normal)] × 100%.

#### 3.8.2. CES-Induced MLE-12 Cells [30]

MLE-12 cells were cultured in 96-well plates using MLE-12 special medium at a density of 3.5 × 10^4^ cells/mL. After 4 h of culturing, the cells were treated with target compounds **9** and **14** at the specified concentrations (0.01, 0.1, and 1 μM) for 12 h. Then, the cells were stimulated using 10% CES for another 24 h. Finally, the treated cells were stimulated using CCK-8 for 2 h at 37 °C, and the absorbance was determined at 450 nm using a microplate spectrophotometer. Solvent-only cells served as negative controls. The cell survival rate was calculated according to the following formula:

$$\mathrm{Cell}\;\mathrm{survival}\;(\%)=\frac{Va}{Vc}\;\times 100\%$$

where *V_a_* is the OD_450_ of the target compounds, and *V_c_* is the OD_450_ of the negative control.

## 4. Conclusions

In this work, we presented an integrated study of the large-scale preparation of cycloastragenol (CAG) and the quality control, isolation, structural elucidation, and activity evaluation of two new impurities. Firstly, a 2.5-kg preparation of cycloastragenol was achieved, and a total of 15 impurities were detected from CAG API for the first time. Among these impurities, compounds **1**, **4**, **9**, **10**, **14**, and **15** were elucidated via spectroscopic analysis, and **2**–**3**, **5**–**8**, and **11**–**13** were putatively identified. Interestingly, two of the new compounds, i.e., compounds **9** and **14**, were rare 10, 19-secocycloartane triterpenoids. In the activity assessment of impurities, the bioassay results demonstrated that the two new compounds **9** and **14** had certain effects on the anti-inflammatory activities against LPS-induced lymphocyte cells and CSE-induced MLE-12 cells. In addition, a plausible structural transformation pathway of the degradation compounds from CAG or AS-IV was proposed. As these compounds did not contain chromophores or possessed weak UV absorption characteristics, we constructively proposed establishing an HPLC-CAD method for carrying out the quality control of the corresponding degradation impurities. The obtained information provides a foundation for further research into CAG and compounds with weak UV absorption characteristics. More importantly, proper guidelines will also be provided on the quality control of APIs and related preparations. Meanwhile, this study may represent an important step toward the development of anti-COPD drugs.

## 5. Patents

An invention patent regarding the work reported in this manuscript has been submitted to protect the methods employed and results identified.

## Figures and Tables

**Table 1:** NMR spectral data of compound **9**.

NO.	δH (J in Hz) ^a^	δC (Type) ^b^	1H-1H COSY	HMBC
1	1.67 (1H, m) 2.42 (1H, m)	35.5 (CH_2_)	H-2	C-10
2	1.64 (1H, m) 1.92 (1H, m)	26.5 (CH_2_)	H-1, 3	-
3	3.68 (1H, d, 4.9)	82.7 (CH)	H-2	C-2, 5, 10, 28
4	-	46.1 (C)	-	-
5	1.46 (1H, m)	61.4 (CH)	H-6	C-6, C-10
6	4.40 (1H, m)	65.9 (CH)	H-5, 7	-
7	1.61 (1H, m) 2.38 (1H, m)	34.6 (CH_2_)	H-6, 8	-
8	2.42 (1H, m)	46.6 (CH)	H-7	-
9	-	35.5 (C)	-	-
10	-	93.2 (C)	-	-
11	1.47 (1H, m) 1.97 (1H, m)	32.8 (CH_2_)	H-12	-
12	1.23 (1H, m) 1.71 (1H, m)	32.4 (CH_2_)	H-11	-
13	-	45.6 (C)	-	-
14	-	45.7 (C)	-	-
15	1.62 (1H, m) 2.04 (1H, m)	46.0 (CH_2_)	H-16	C-14, 17, 30
16	5.02 (1H, m)	73.0 (CH)	H-15, 17	C-14
17	2.43 (1H, m)	57.4 (CH)	H-16	C-13, 16, 18, 20, 21, 22
18	1.34 (3H, s)	19.0 (CH_3_)	-	C-12, 13, 14, 17
19	1.40 (3H, s)	35.8 (CH_3_)	-	C-8, 9, 10, 11
20	-	87.1 (C)	-	-
21	1.33 (3H, s)	28.6 (CH_3_)	-	C-17, 20, 22
22	1.63 (1H, m) 3.08 (1H, m)	34.8 (CH_2_)	H-23	C-20, 21, 23
23	2.09 (1H, m) 2.27 (1H, m)	26.5 (CH_2_)	H-22, 24	-
24	3.89 (1H, dd, 8.7, 4.4)	81.6 (CH)	H-23	C-25
25	-	71.4 (C)	-	-
26	1.30 (3H, s)	27.0 (CH_3_)	-	C-24, 25, 26
27	1.57 (3H, s)	28.1 (CH_3_)	-	C-24, 25, 27
28	1.41 (3H, s)	23.1 (CH_3_)	-	C-3, 4, 5, 29
29	1.32 (3H, s)	26.1 (CH_3_)	-	C-3, 4, 5, 28
30	0.97 (3H, s)	19.8 (CH_3_)	-	C-8, 13, 14, 15

^a^ Spectrum recorded at 400 MHz in C5D5N. ^b^ Spectrum recorded at 100 MHz in C5D5N.

**Table 2:** NMR spectral data of compound **14**.

NO.	*δ*_H_ (*J* in Hz) ^a^	*δ*_C_ (Type) ^b^	^1^H-^1^H COSY	HMBC
1	5.59 (1H, brs)	115.2 (CH)	H-2	C-3, 9, 10
2	2.42 (1H, m) 2.66 (1H, m)	33.4 (CH_2_)	H-1, 3	C-1, 3, 4, 10
3	3.80 (1H, dd, 9.6, 6.0)	72.6 (CH)	H-2	C-28, 29
4	-	40.1 (C)	-	-
5	-	142.3 (C)	-	-
6	5.59 (1H, brs)	119.6 (CH)	H-7	C-5
7	2.03 (1H, m) 2.40 (1H, m)	25.2 (CH_2_)	H-6, 8	C-5, 6, 8
8	2.06 (1H, m)	42.2 (CH)	H-7	C-6, 7, 9, 10, 19
9	-	37.2 (C)	-	-
10	-	139.7 (C)	-	-
11	1.69 (1H, m) 2.04 (1H, m)	31.0 (CH_2_)	H-12	-
12	1.46 (1H, m) 1.82 (1H, m)	31.3 (CH_2_)	H-11	-
13	-	46.9 (C)	-	-
14	-	47.8 (C)	-	-
15	1.59 (1H, m) 2.06 (1H, m)	45.4 (CH_2_)	H-16	C-14, 17, 30
16	5.07 (1H, dd, 13.7, 7.0)	73.3 (CH)	H-15, 17	C-13
17	2.48 (1H, m)	56.8 (CH)	H-16	C-13, 16, 18, 20, 21, 22
18	1.41 (3H, s)	19.2 (CH_3_)	-	C-12, 13, 14, 17
19	1.05 (3H, s)	32.2 (CH_3_)	-	C-8, 9, 10, 11
20	-	87.2 (C)	-	-
21	1.34 (3H, s)	28.6 (CH_3_)	-	C-17, 20, 22
22	1.65 (1H, m) 3.07 (1H, dd, 21.2, 10.5)	34.8 (CH_2_)	H-23	C-20, 21, 23
23	2.09 (1H, m) 2.29 (1H, m)	26.5 (CH_2_)	H-22, 24	-
24	3.88 (1H, dd, 9.1, 4.5)	81.6 (CH)	H-23	C-25
25	-	71.4 (C)	-	-
26	1.29 (3H, s)	27.0 (CH_3_)	-	C-24, 25, 27
27	1.53 (3H, s)	28.1 (CH_3_)	-	C-24, 25, 26
28	1.41 (3H, s)	23.8 (CH_3_)	-	C-3, 4, 5, 29
29	1.13 (3H, s)	20.0 (CH_3_)	-	C-3, 4, 5, 28
30	0.92 (3H, s)	18.1 (CH_3_)	-	C-8, 13, 14, 15

^a^ Spectrum recorded at 400 MHz in C5D5N. ^b^ Spectrum recorded at 100 MHz in C5D5N.

**Table 3:** Retention times and MS data used in the identification of 15 compounds.

NO.	*t_R_* (min)	Formula	CAS	*m*/*z*				Fragment Ion
				[M+H]^+^	[M+Na]^+^	[M+HCOOH-H]^−^	[M-H]^−^	
1 *	9.83	C_35_H_58_O_9_	86541-83-5		645.3965 (1.2)	667.4053 (0.6)		125.0960, 143.1065, 279.0928[Xyl+3Ac]^+^, 419.3302, 437.3408, 455.3512, 473.3618
2	11.99	C_36_H_60_O_10_	86764-12-7		675.4068 (1.6)	697.4161 (0.3)	651.4112 (0.6)	125.0961, 143.1065, 224.1277[Glu+Ac+H]^+^, 419.3302, 437.3408, 455.3513, 473.3618, 645.3959[M+2H_2_O-Ac]^+^
3	21.34	C_35_H_58_O_9_	1638800-96-0		645.3967 (0.9)	667.4056 (0.1)	621.4002 (0.1)	143.1065, 297[Xyl+H_2_O+3Ac]^+^, 419.3302, 437.3408, 455.3512
4 *	22.36	C_30_H_50_O_5_	86541-79-9		513.3356 (1.1)	535.3638 (0.5)	489.3586 (1.2)	125.0962, 143.1067, 419.3307, 437.3414, 455.3519, 473.3627, 981.7380[2M+H]^+^
5	25.92	C_30_H_46_O_5_	-		509.3243 (1.1)	531.3329 (1.3)	485.3275 (1.6)	125.0964, 143.1068, 437.3419, 469.3317 [M-H_2_O]^+^
6	26.42	C_32_H_52_O_6_	80604-30-4		555.3663 (1.2)			125.0964, 143.1059, 419.3312, 437.3419, 455.3524, 473.3631, 495.3450[M-H-2H_2_O]^+^
7	26.96	C_30_H_48_O_5_	2305765-07-3		511.3399 (0.9)	533.3478 (0)		107.0860, 125.0964, 143.1068, 435.3262, 453.3366, 471.3473[M+H-H_2_O]^+^
8	29.80	C_30_H_46_O_5_	145497-67-2		509.3246 (1.7)	531.3326 (0.7)	485.3271 (0.8)	107.0860, 125.0964, 419.1069, 437.3421, 455.3525, 469.3319[M+H-H_2_O^]+^
9 *	33.76	C_30_H_50_O_5_	-	491.3734 (0.6)	513.3557 (1.3)	535.3636 (0.2)		107.0860, 125.0963, 143.1068, 419.3311, 437.3418, 455.3523, 473.3629
10 *	34.11	C_32_H_52_O_6_	199680-24-5	533.3840 (0.5)	555.3659 (0.5)	577.3743 (0.5)		107.0859, 125.0963, 143.1068, 419.3310, 437.3416, 455.3521, 497.3625[M+H-2H_2_O]^−^
11	37.40	C_30_H_48_O_4_	1807473-07-9		495.3448 (0.6)	517.3528 (0.2)		107.0860, 125.0963, 143.1068, 419.3311, 437.3417, 455.3522
12	38.47	C_30_H_48_O_4_	819821-43-7	473.3630 (0)	495.3450 (0)			107.0860, 125.0963, 143.1068, 419.3311, 437.3417, 455.3522
13	41.40	C_30_H_50_O_5_	97682-74-1		513.3557 (1.4)	535.3644 (1.7)	489.3590 (2.0)	419.3312, 437.3420, 453.3368[M-H-2H_2_O^+^]^+^
14 *	43.58	C_30_H_48_O_4_	-	473.3627 (0.4)	495.3449 (0.8)	517.3529 (0)		107.0860, 125.0963, 143.1068, 419.3311, 437.3418, 455.3523
15 *	46.36	C_32_H_52_O_6_	1252686-32-0		555.3660 (0.7)	577.3742 (0.3)		107.0860, 125.0963, 143.1068, 419.3311, 437.3418, 455.3523, 479.3523[M+H-3H_2_O]^+^, 515.3735[M+H-H_2_O]^+^

* Refers to the main isolated impurities with area normalized contents greater than 0.05% from Fraction B. () Refers to the accurate mass error (ppm).

## Data Availability

Data supporting reported results can be found in the Supplementary Materials part, https://www.mdpi.com/article/10.3390/molecules28176382/s1.

## Supplementary Materials

The following are available online at https://www.mdpi.com/article/10.3390/molecules28176382/s1: Figures S1–S11: The positive HRESIMS and NMR spectra of compound **9**; Figures S12–S23: The positive HRESIMS and NMR spectra of compound **14**; Table S1: ^13^C NMR spectral data of compounds **9** and **14** and CAG; Table S2: Anti-inflammatory activity of compound **9** against LPS-induced lymphocyte cells; Table S3: Anti-inflammatory activity of compound **14** against LPS-induced lymphocyte cells; Table S4: Anti-inflammatory activity of compound **9** and **14** against CSE-induced MLE-12 cells; Figures S24–S38: ESI-MS/MS spectra of compound **1**–**15**.

## Sample Availability

Samples of the compounds **1**, **4**, **9**, **10**, **14**, and **15** are available from the authors.
