# Supplementary material for: Using UPLC-LTQ-Orbitrap-MS and HPLC-CAD to Identify Impurities in Cycloastragenol, Which Is a Pre-Clinical Candidate for COPD
Source: Molecules. 2023 Aug 31;28(17):6382. doi: 10.3390/molecules28176382 (PMC10489802; doi:10.3390/molecules28176382)
Supplement: Supplementary file 1 [file molecules-28-06382-s001.zip › molecules-2553469-supplementary.pdf]

Supporting Information

Using UPLC-LTQ-Orbitrap-MS and HPLC-CAD to Identify Impurities in Cycloastragenol, which is a Pre-Clinical Candidate for COPD

Feng Zhu[1], Xiao Zhang [1,2], Bing-Yuan Du[1], Xiang-Xia Zhu[1], Gui-Fang Zhao[1], Ying Sun[1,2], Qing-Qiang Yao[3], Hong-Bao Liang[1,2], Jing-Chun Yao[1], Zhong Liu[1], Gui-Min Zhang[1,2,*], and Guo-Fei Qin[1,*]

[1] State Key Laboratory of Integration and Innovation of Classic Formula and Modern Chinese Medicine, Lunan Pharmaceutical Group Co. Ltd., Linyi 273400, China; zhufeng0716@163.com (F.Z.); szyzhangxiao@163.com (X.Z.); dubingyuanyuan@126.com (B.-Y.D.); zhuxiangxia1986@163.com (X.-X.Z.); zhaoguifang2000@163.com (G.-F.Z.); sunying1982@126.com (Y.S.); lianghongbao1985@163.com (H.-B.L.); yaojingchun@lunan.cn (J.-C.Y.); clevertree@163.com (Z.L.)

[2] College of Pharmacy, Shandong University of Traditional Chinese Medicine, Jinan 250355, China

[3] Jining Medical University, Jining 272067, China; yao_imm@163.com

* Correspondence: lunanzhangguimin@163.com (G.-M.Z.); qinguofei@126.com (G.-F.Q.); Tel.: +86-539-503-0319 (G.-F.Q.); Fax: +86-539-503-0800

Table of Contents

Figure S1 The positive HRESIMS spectrum of compound **9** **S3**
Figure S2 ^{1}H NMR (400 MHz, C5D5N) spectrum of compound **9** **S4**
Figure S3 Amplificatory ^{1}H NMR spectrum of compound **9** **S5**
Figure S4 ^{13}C NMR (100 MHz, C5D5N) spectrum of compound **9** **S6**
Figure S5 Amplificatory ^{13}C NMR spectrum of compound **9** **S7**
Figure S6 DEPT spectrum of compound **9** **S8**
Figure S7 HMQC spectrum of compound **9** **S9**
Figure S8 Amplificatory HMQC spectrum of compound **9** **S10**
Figure S9 ^{1}H-^{1}H COSY spectrum of compound **9** **S11**
Figure S10 Key HMBC spectrum of compound **9** **S12**
Figure S11 Amplificatory HMBC spectrum of compound **9** **S13**
Figure S12 The positive HRESIMS spectrum of compound **14** **S14**
Figure S13 ^{1}H NMR (400 MHz, C_5D_5N) spectrum of compound **14** **S15**
Figure S14 Amplificatory ^{1}H NMR spectrum of compound **14** **S16**
Figure S15 ^{13}C NMR (100 MHz, C_5D_5N) spectrum of compound **14** **S17**
Figure S16 Amplificatory ^{13}C NMR spectrum of compound **14** **S18**
Figure S17 DEPT spectrum of compound **14** **S19**
Figure S18 HMQC spectrum of compound **14** **S20**

Figure S19 Amplificatory HMQC spectrum of compound **14** S21
Figure S20 ^{1}H-^{1}H COSY spectrum of compound **14** S22
Figure S21 Key HMBC spectrum of compound **14** S23
Figure S22 Amplificatory HMBC spectrum of compound **14** (A) S24
Figure S23 Amplificatory HMBC spectrum of compound **14** (B) S25
Table S1 ^{13}C NMR spectral data of compounds **9**, **14** and CAG S26
Table S2 Anti-inflammatory of compound **9** activities against LPS-induced lymphocyte cells S27
Table S3 Anti-inflammatory of compound **14** activities against LPS-induced lymphocyte cells S27
Table S4 Anti-inflammatory of compound **9** and **14** activities against CSE-induced MLE-12 cells S28
Figure S24 Positive ions (A) and negative ions (B) ESI-MS/MS spectra of compound **1** S29
Figure S25 Positive ions (A) and negative ions (B) ESI-MS/MS spectra of compound **2** S30
Figure S26 Positive ions (A) and negative ions (B) ESI-MS/MS spectra of compound **3** S31
Figure S27 Positive ions (A) and negative ions (B) ESI-MS/MS spectra of compound **4** S32
Figure S28 Positive ions (A) and negative ions (B) ESI-MS/MS spectra of compound **5** S33
Figure S29 Positive ions ESI-MS/MS spectra of compound **6** S34
Figure S30 Positive ions (A) and negative ions (B) ESI-MS/MS spectra of compound **7** S35
Figure S31 Positive ions (A) and negative ions (B) ESI-MS/MS spectra of compound **8** S36
Figure S32 Positive ions (A) and negative ions (B) ESI-MS/MS spectra of compound **9** S37
Figure S33 Positive ions (A) and negative ions (B) ESI-MS/MS spectra of compound **10** S38
Figure S34 Positive ions ESI-MS/MS spectra of compound **11** S39
Figure S35 Positive ions ESI-MS/MS spectra of compound **12** S40
Figure S36 Positive ions (A) and negative ions (B) ESI-MS/MS spectra of compound **13** S41
Figure S37 Positive ions (A) and negative ions (B) ESI-MS/MS spectra of compound **14** S42
Figure S38 Positive ions (A) and negative ions (B) ESI-MS/MS spectra of compound **15** S43

**Sample Name**	W200994-0003	**Position**	P1-B5	**Instrument Name**	Instrument 1	**User Name**	
**Inj Vol**	1	**InjPosition**		**SampleType**	Sample	**IRM Calibration Status**	Success
**Data Filename**	W200994-0003.d	**ACQ Method**	wss-isocratic elutio	**Comment**		5 **Acquired Time**	7/2/2020 4:30:25 PM

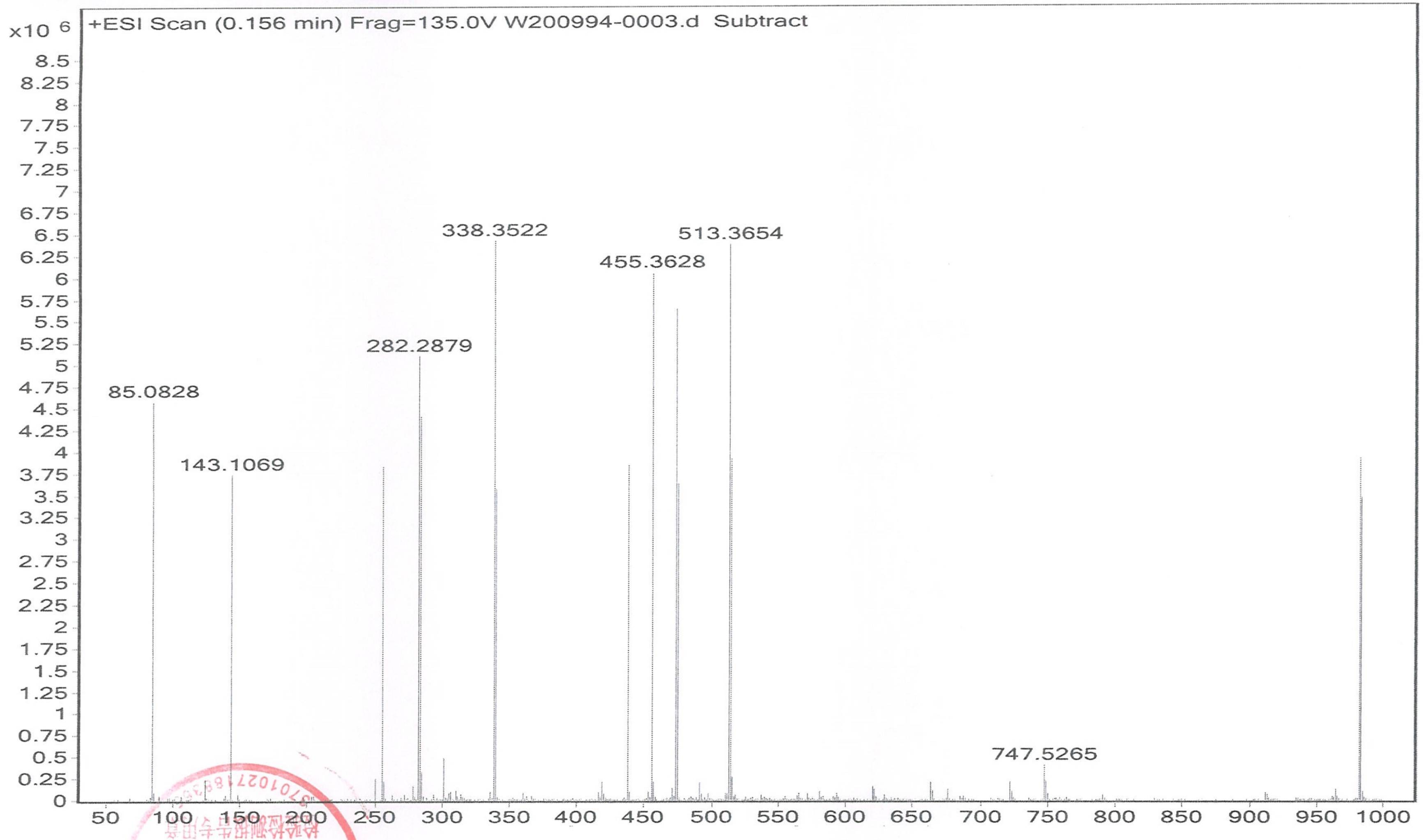

山东省分析测试中心

Figure S1 The positive HRESIMS spectrum of compound 9

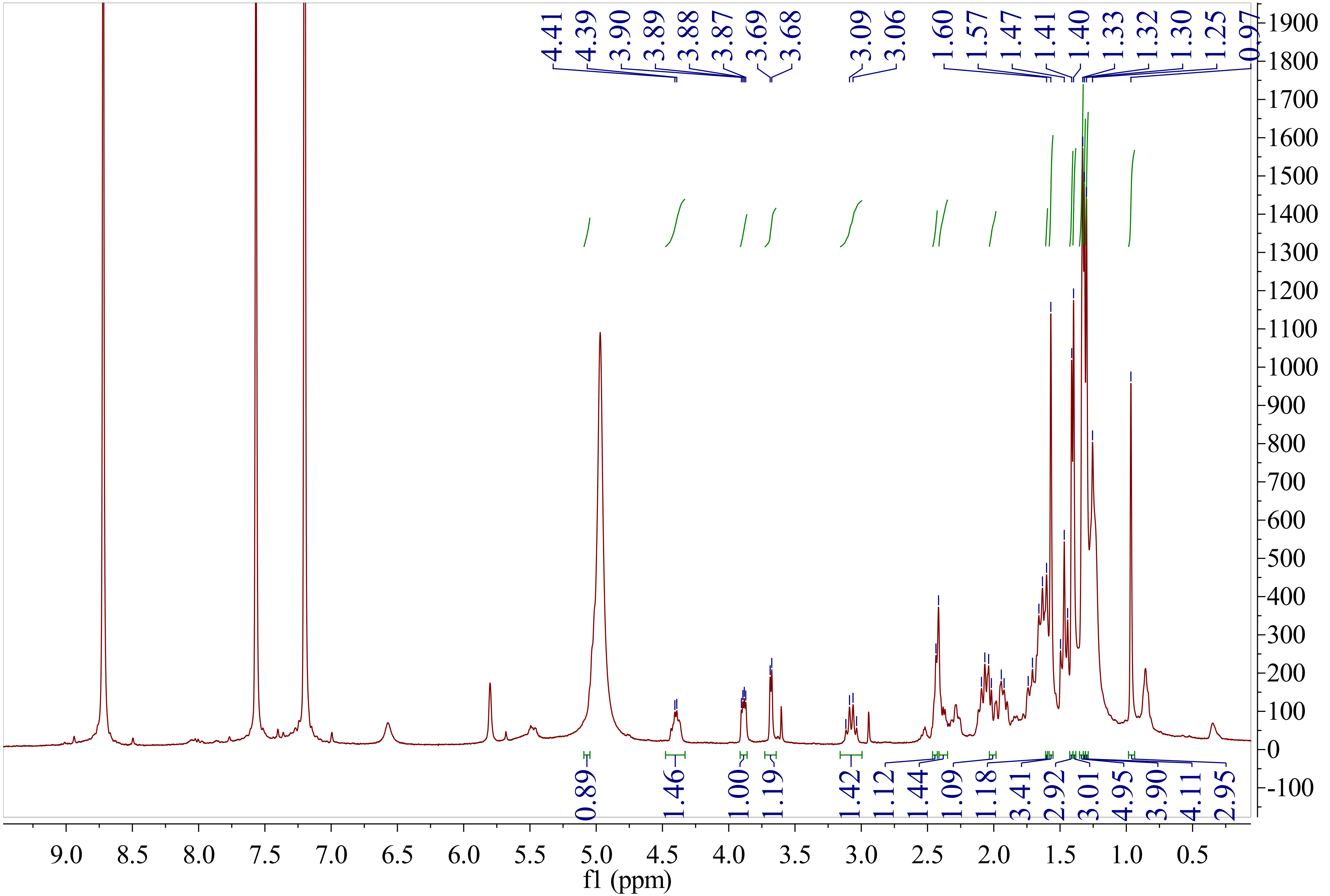

Figure S2 ^{1}H NMR (400 MHz, C_5D_5N) spectrum of compound 9

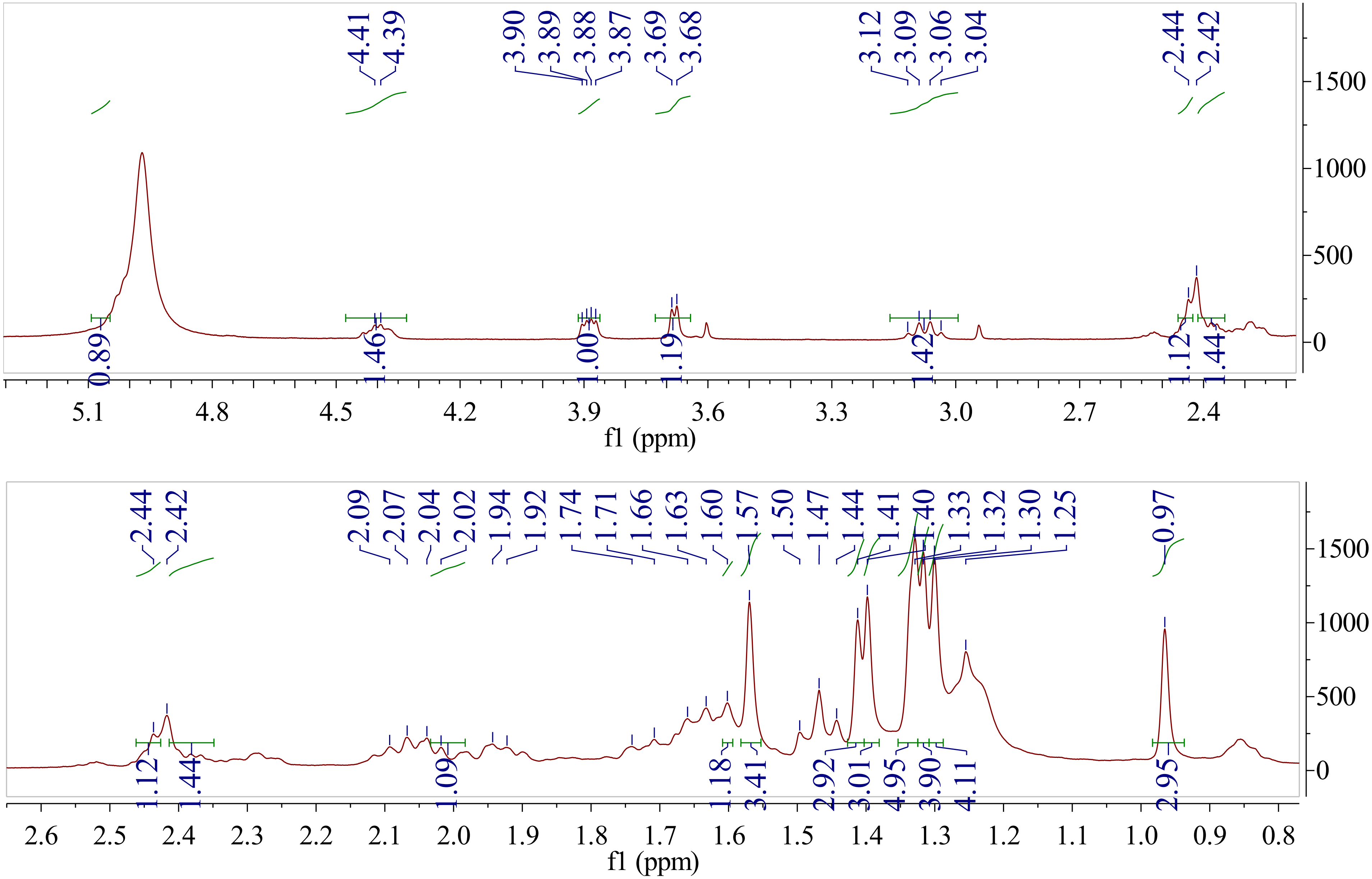

Figure S3 Amplificatory ^{1}H NMR spectrum of compound 9

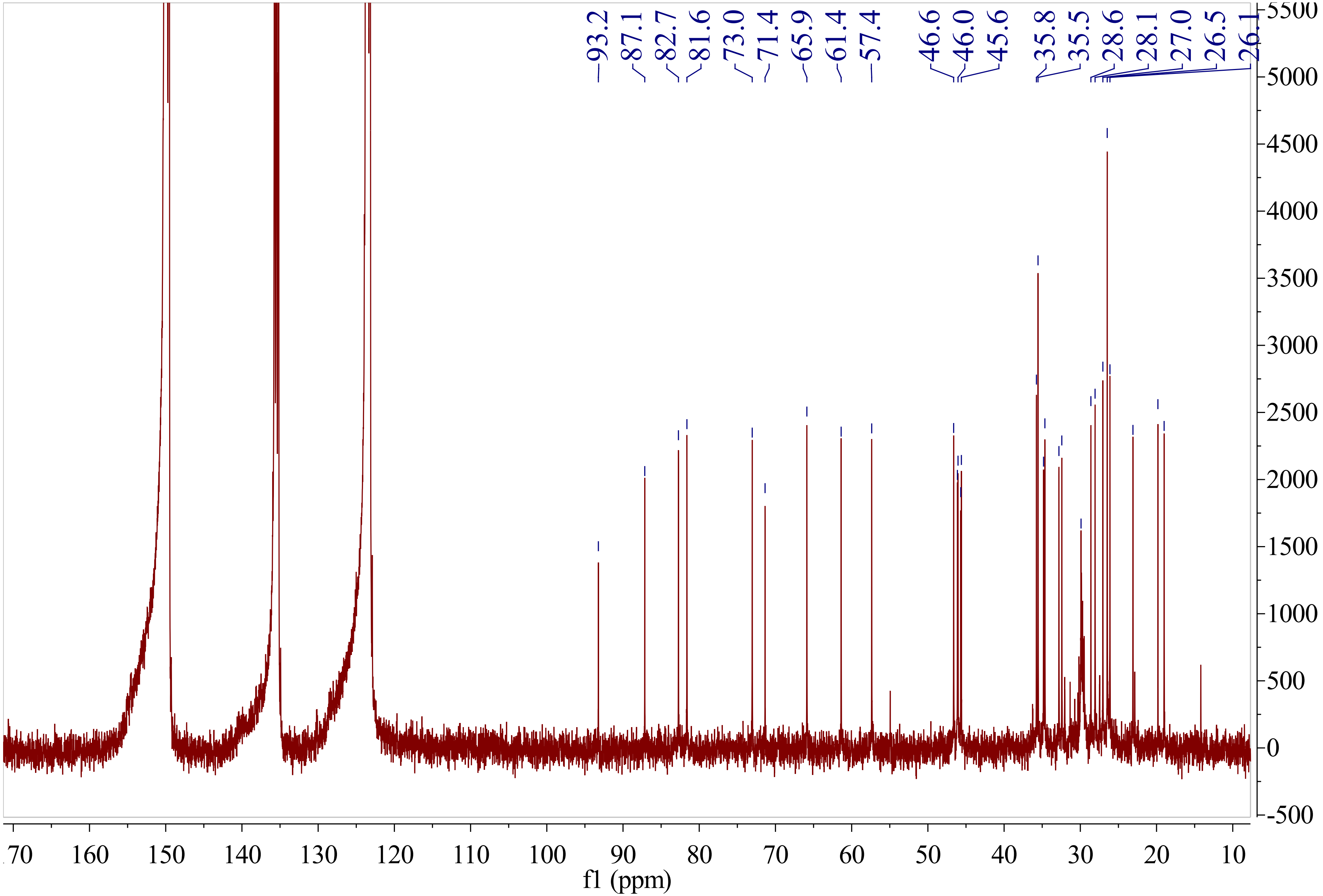

Figure S4 ^{13}C NMR (100 MHz, C_5D_5N) spectrum of compound 9

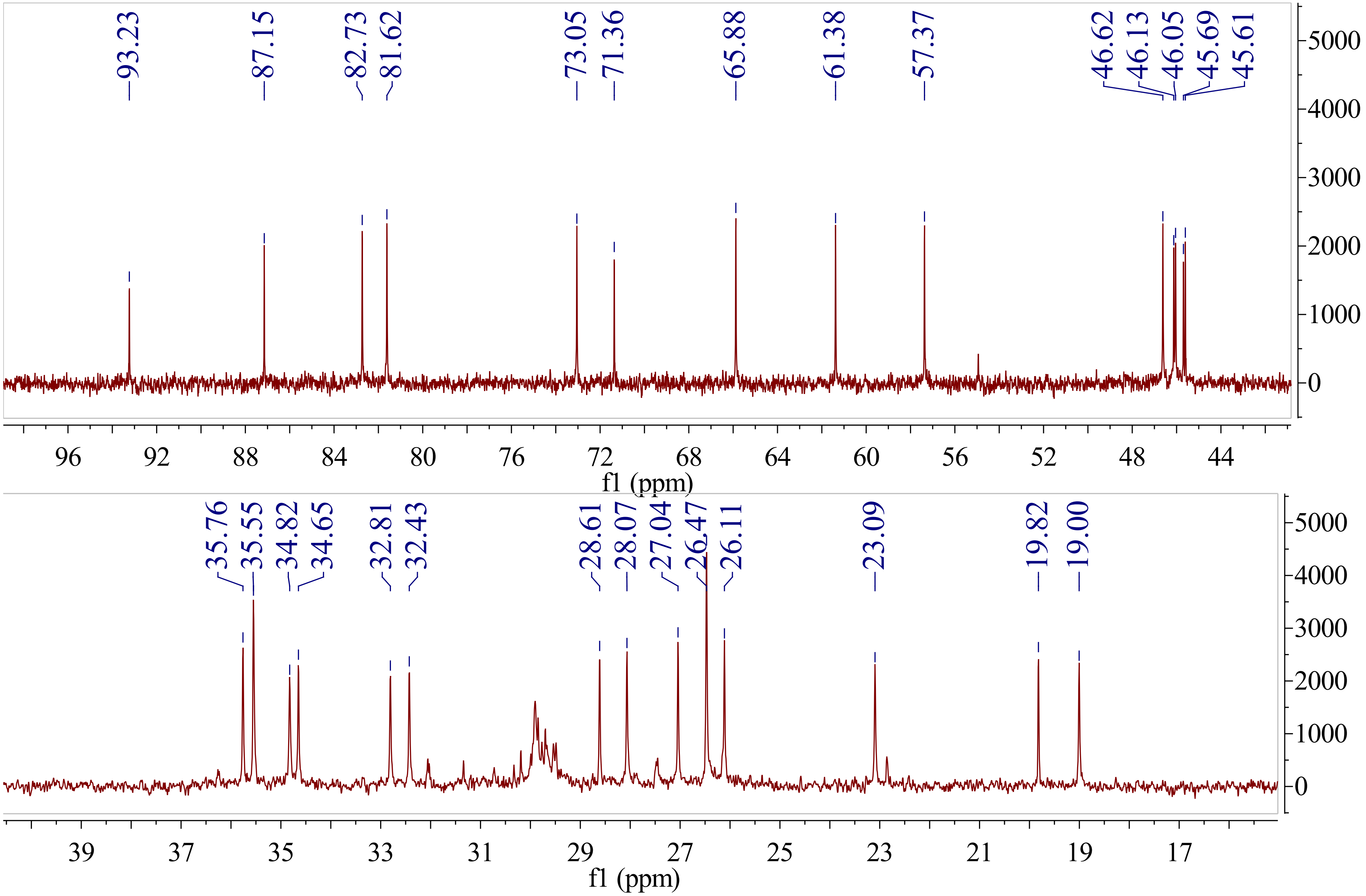

Figure S5 Amplificatory ^{13}C NMR spectrum of compound 9

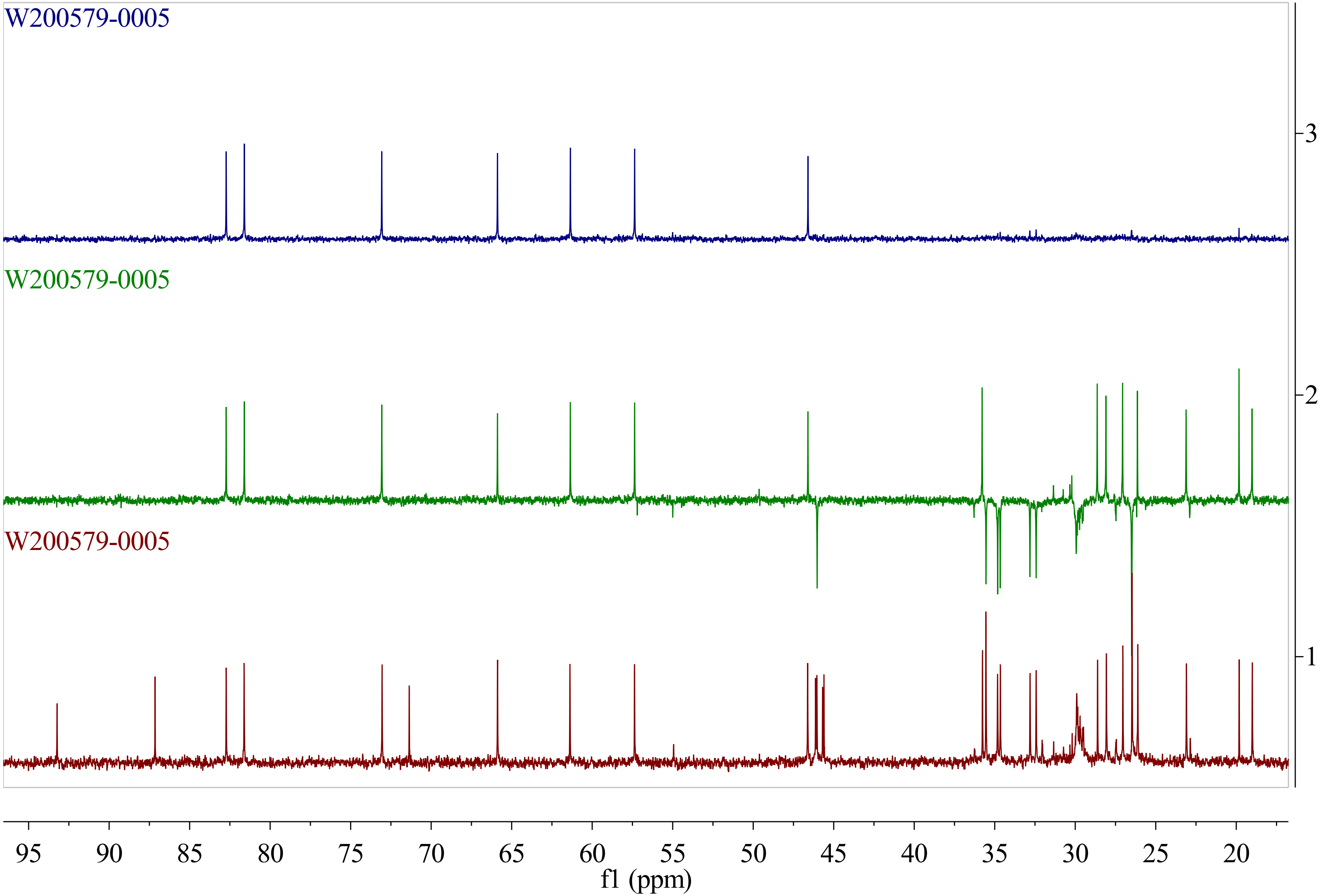

Figure S6 DEPT spectrum of compound 9

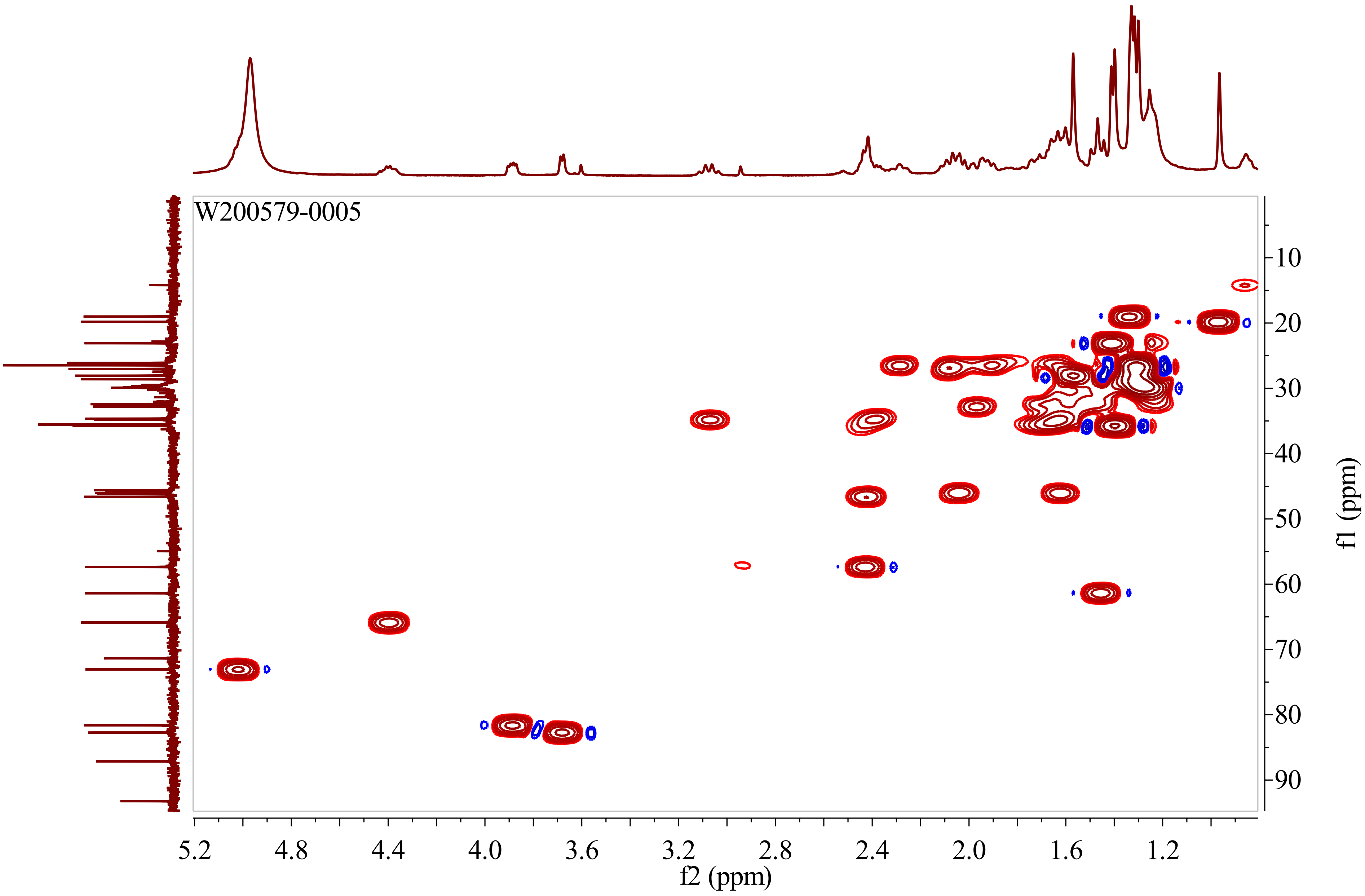

Figure S7 HMQC spectrum of compound 9

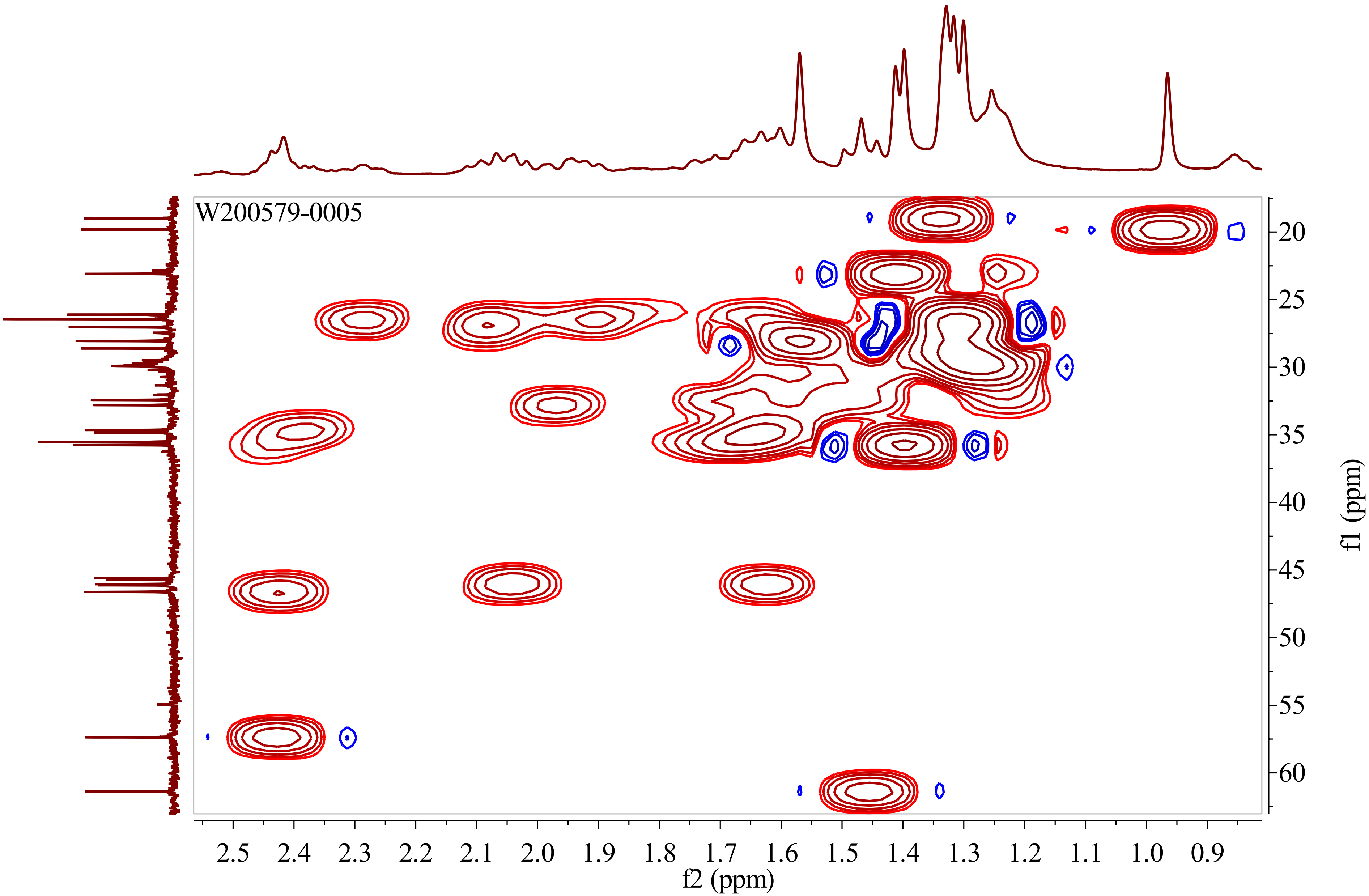

Figure S8 Amplificatory HMQC spectrum of compound 9

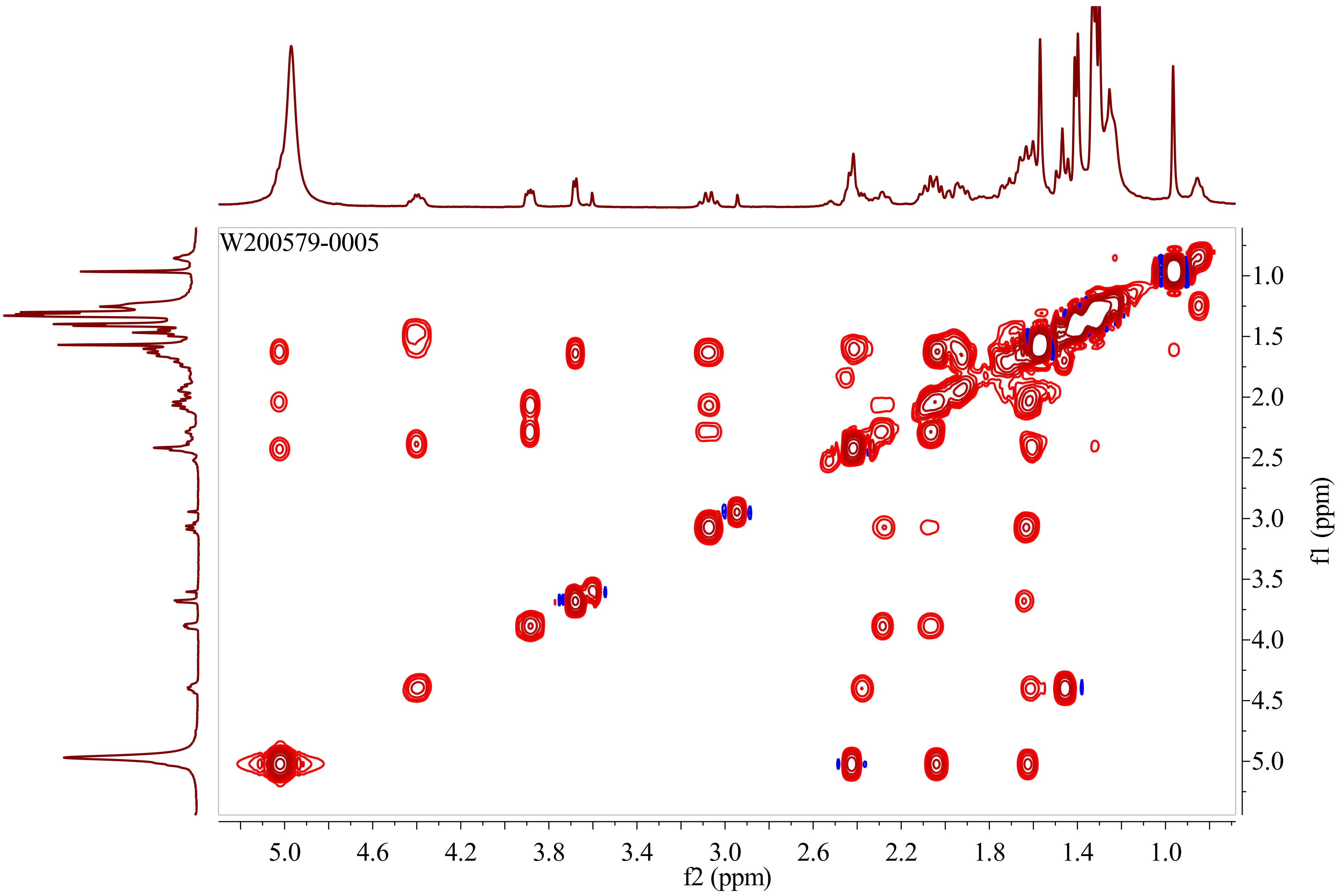

Figure S9 ^{1}H-^{1}H COSY spectrum of compound 9

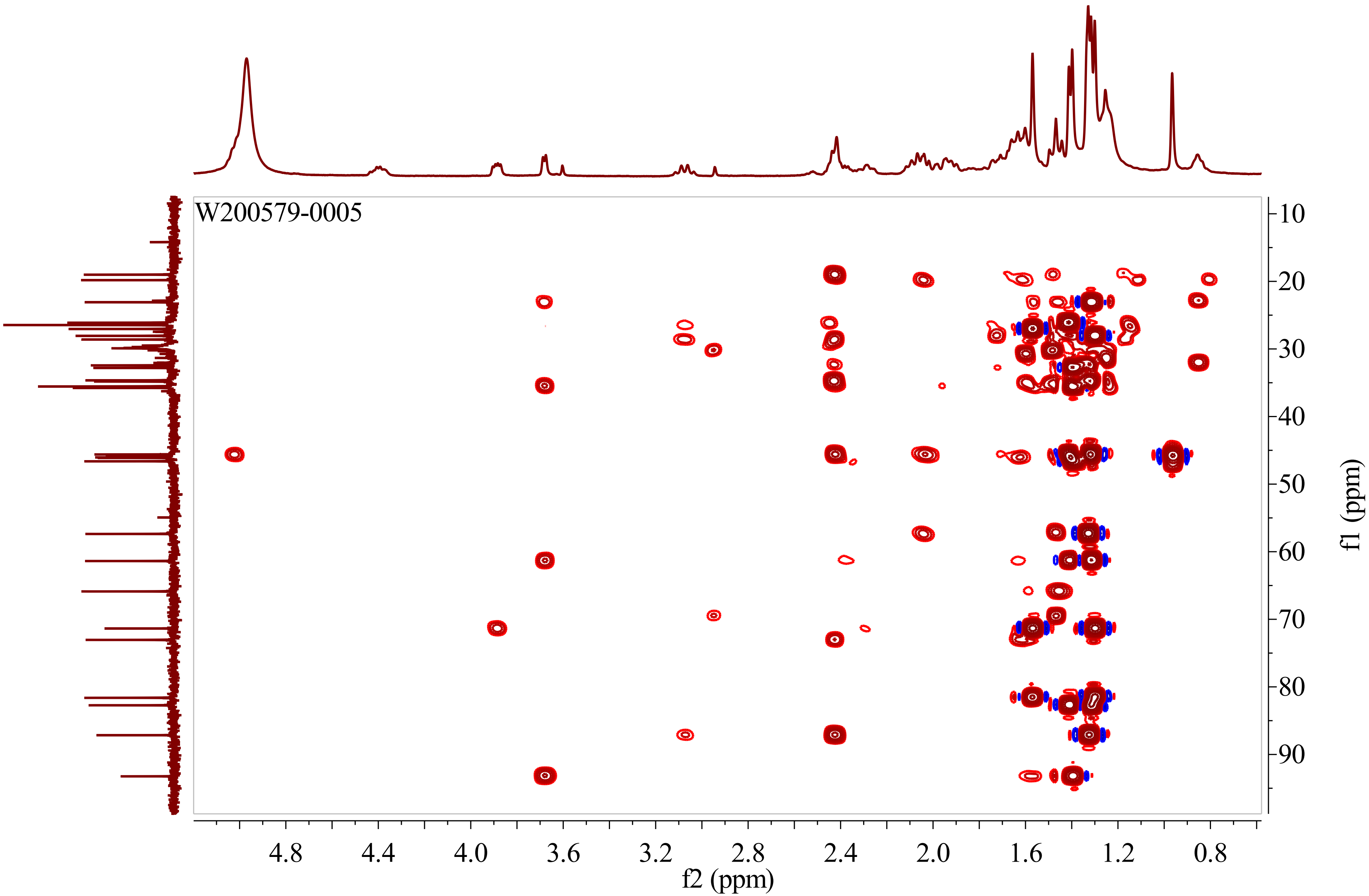

Figure S10 Key HMBC spectrum of compound 9

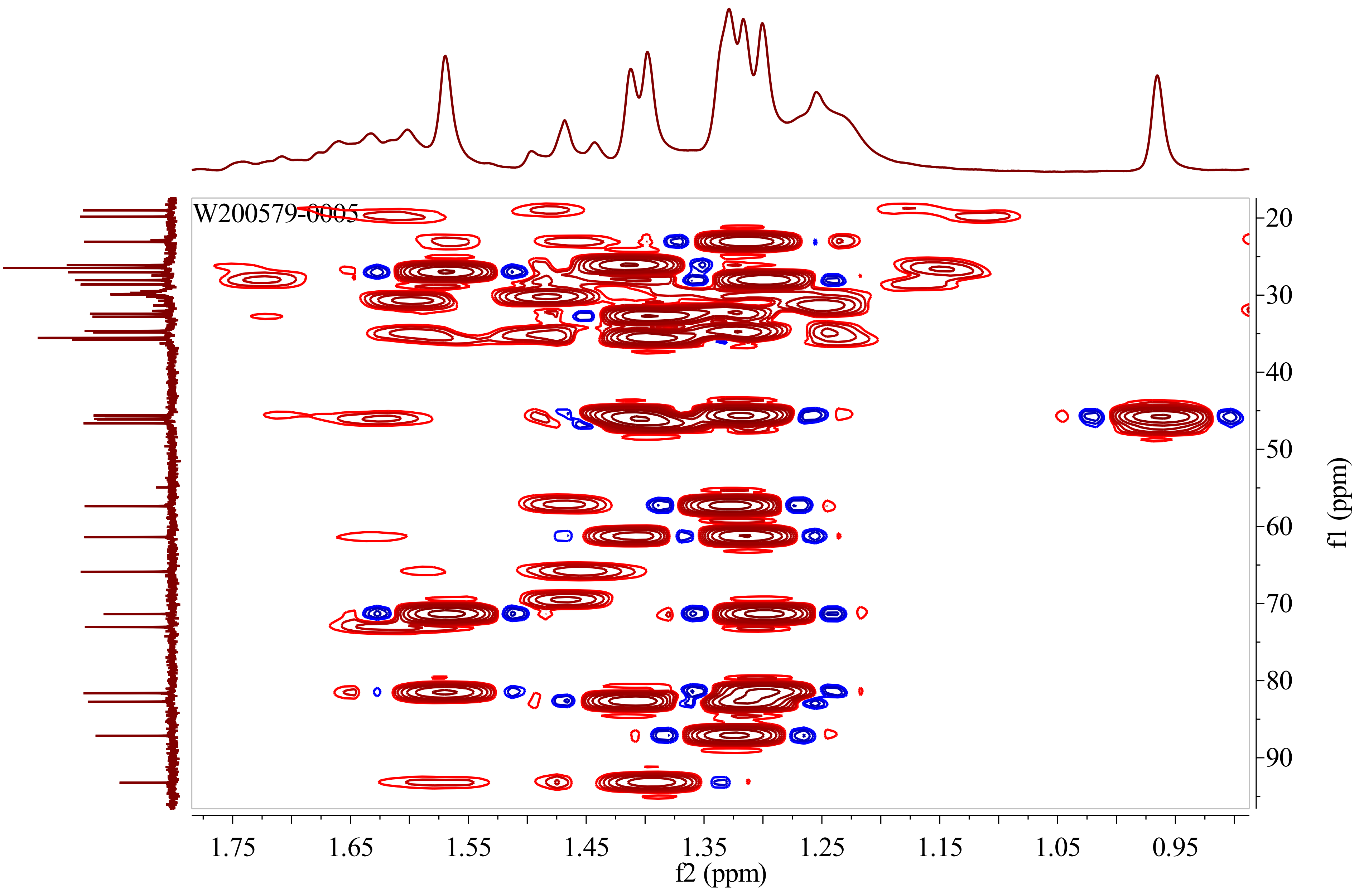

Figure S11 Amplificatory HMBC spectrum of compound 9

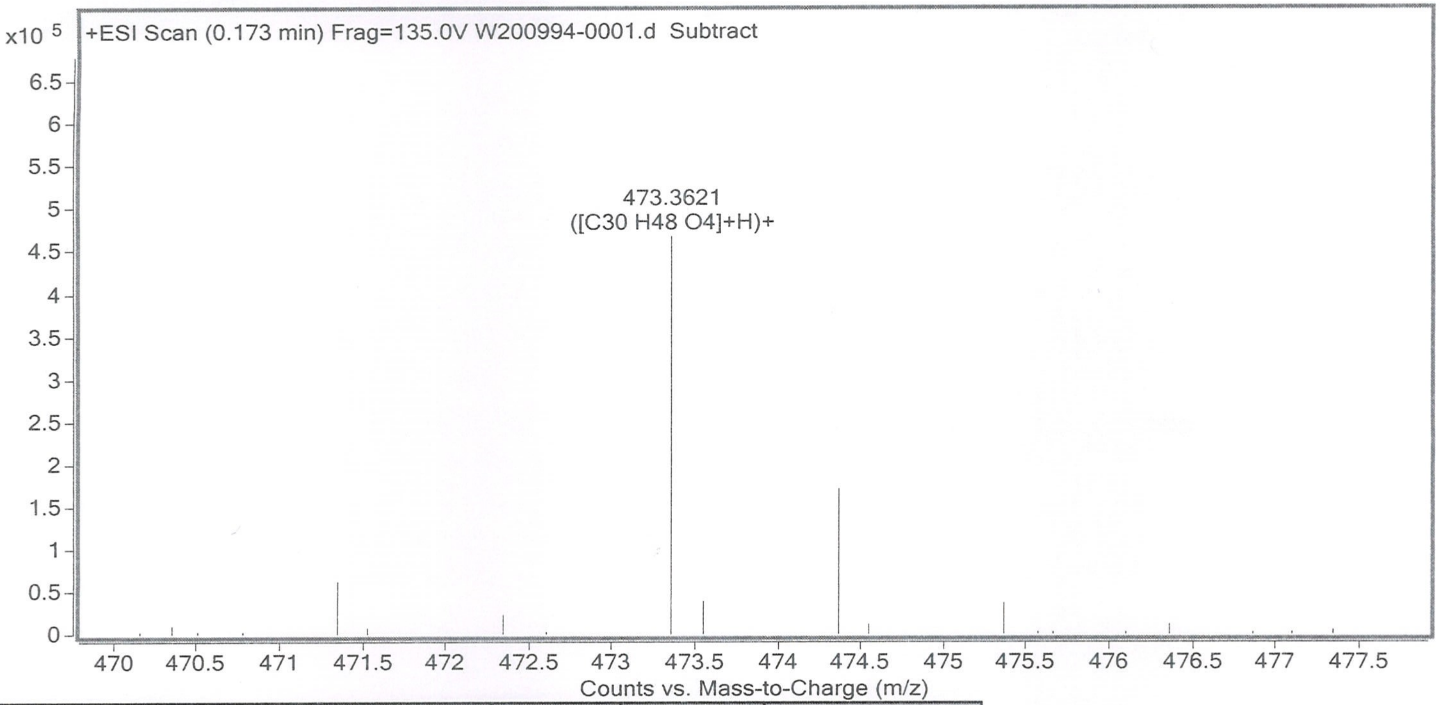

Name	Formula	Species	m/z	Score	Diff (ppm)
	C30 H48 O4	(M+H)+	473.3621	94.9	1.81

m/z	Score (iso. abund)	Score (mass)	Score (MS)	Score (MFG)	Score (iso. spacing)	Height
473.3621	94.39	96.67	94.9	94.9	91.98	467131.1

Height (Calc)	Height Sum% (Calc)	Height % (Calc)	m/z (Calc)	Diff (mDa)	Height	Height %	Height Sum %	m/z	Diff (ppm)
488321.1	71.4	100	473.3625	0.4	467131.1	100	68.3	473.3621	0.83
161942.5	23.7	33.2	474.3659	1.5	169396.9	36.3	24.8	474.3644	3.24
30009.4	4.4	6.1	475.369	2.2	37145.1	8	5.4	475.3668	4.69
4019.9	0.6	0.8	476.3719	5.5	10620	2.3	1.6	476.3664	11.6

Figure S12 The positive HRESIMS spectrum of compound 14

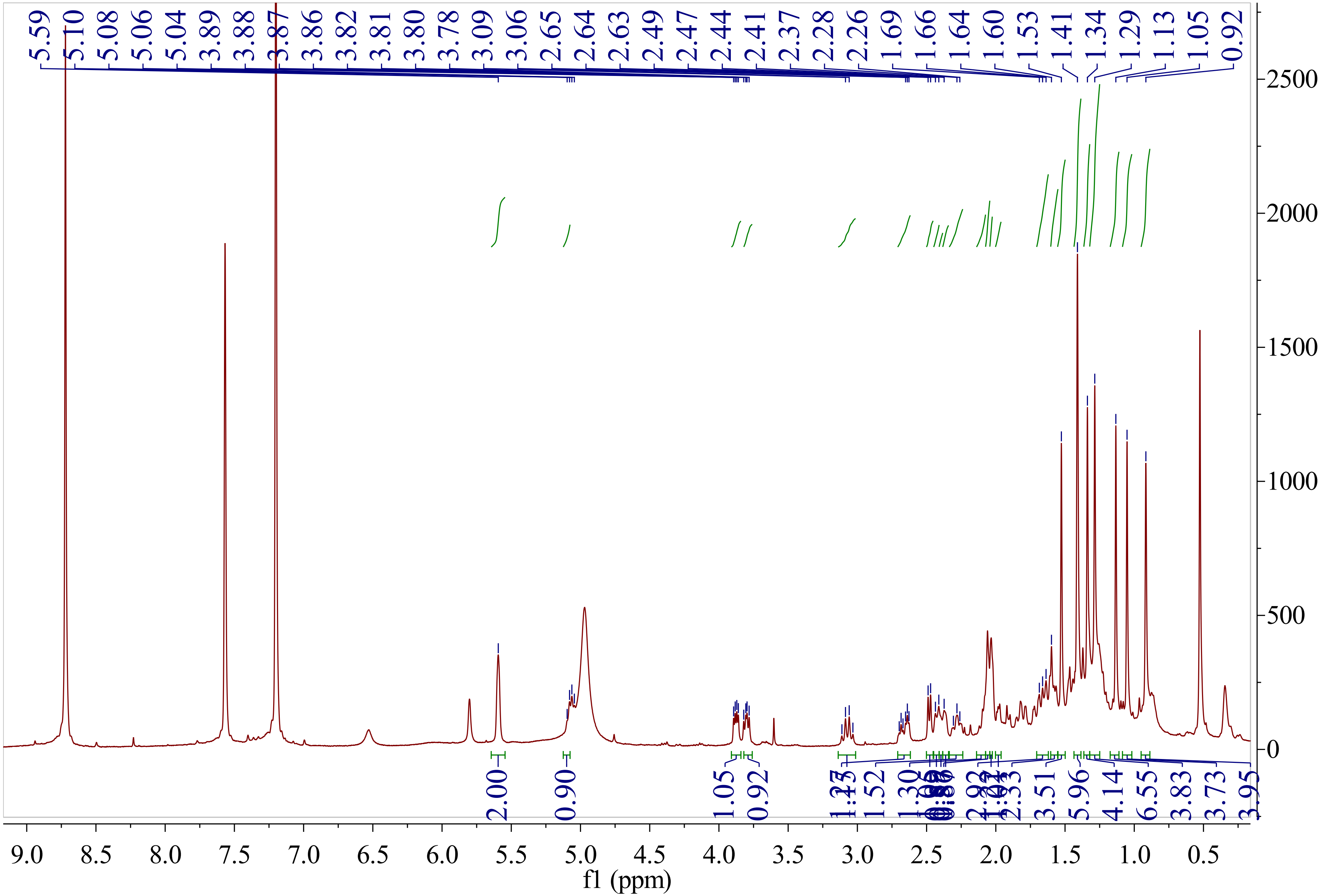

Figure S13 ¹H NMR (400 MHz, C_5D_5N) spectrum of compound 14

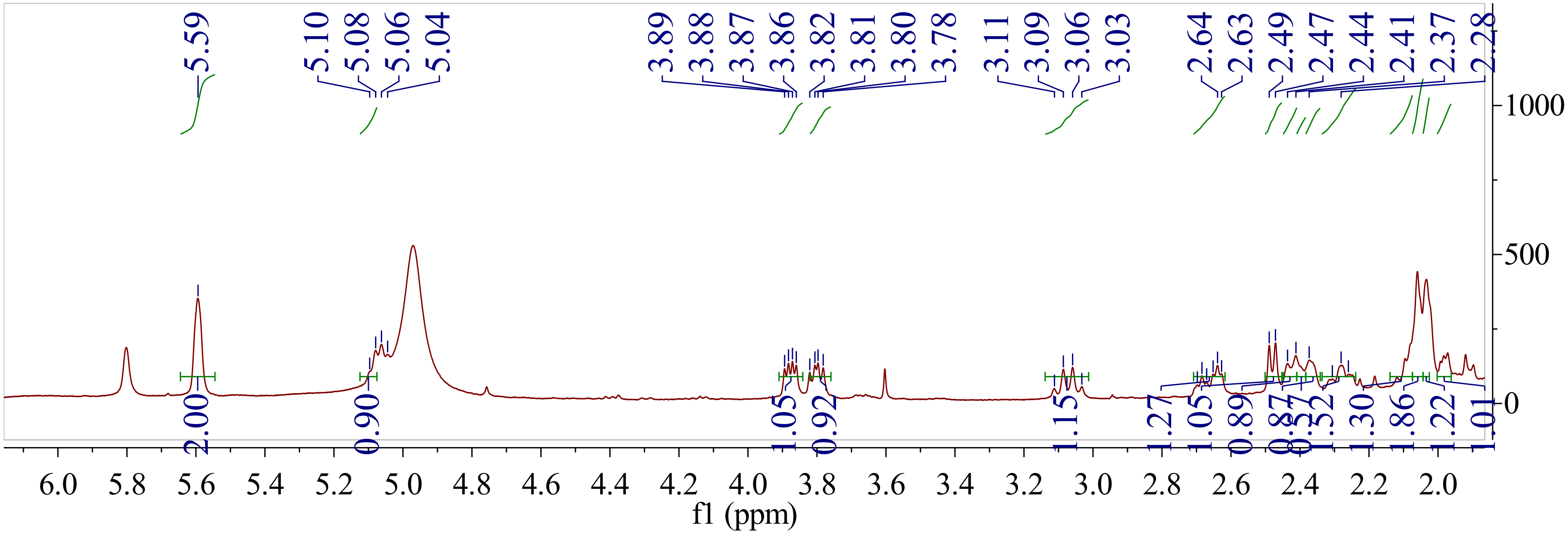

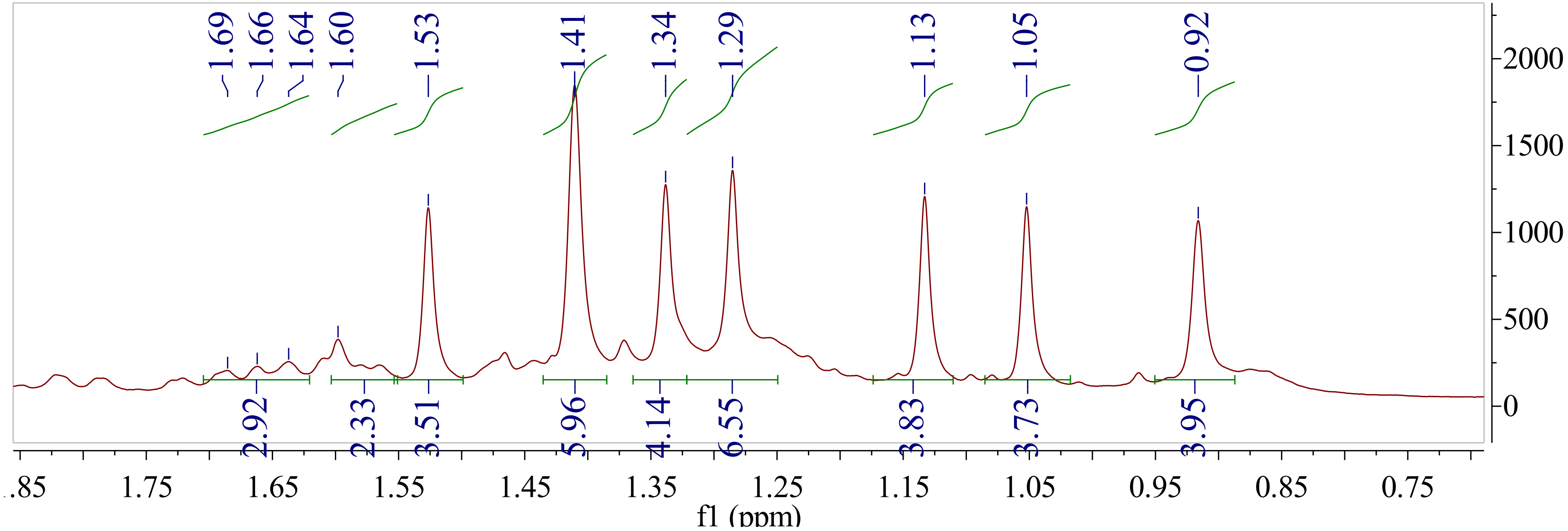

Figure S14 Amplificatory ^{1}H NMR spectrum of compound 14

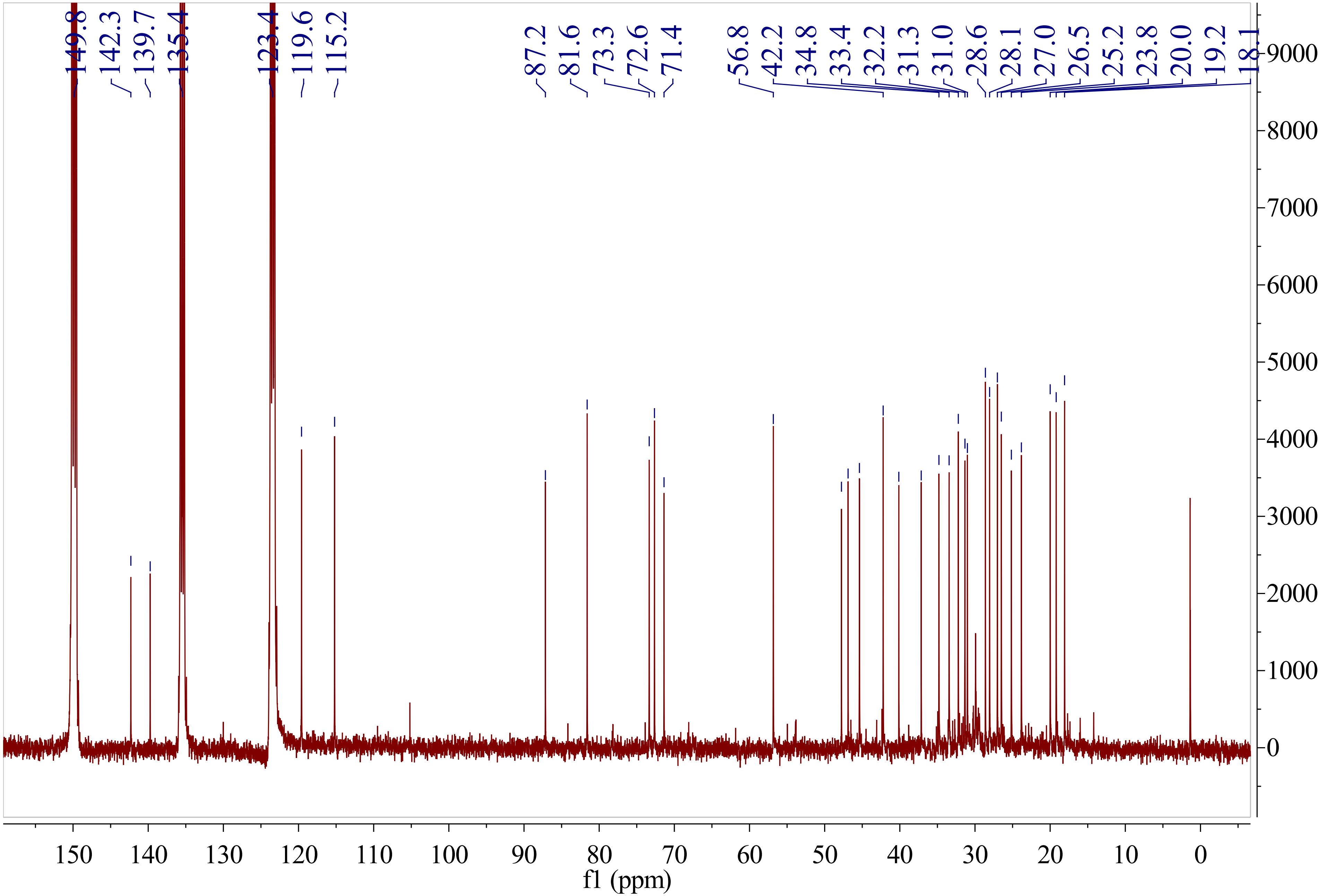

Figure S15 ^{13}C NMR (100 MHz, C_5D_5N) spectrum of compound 14

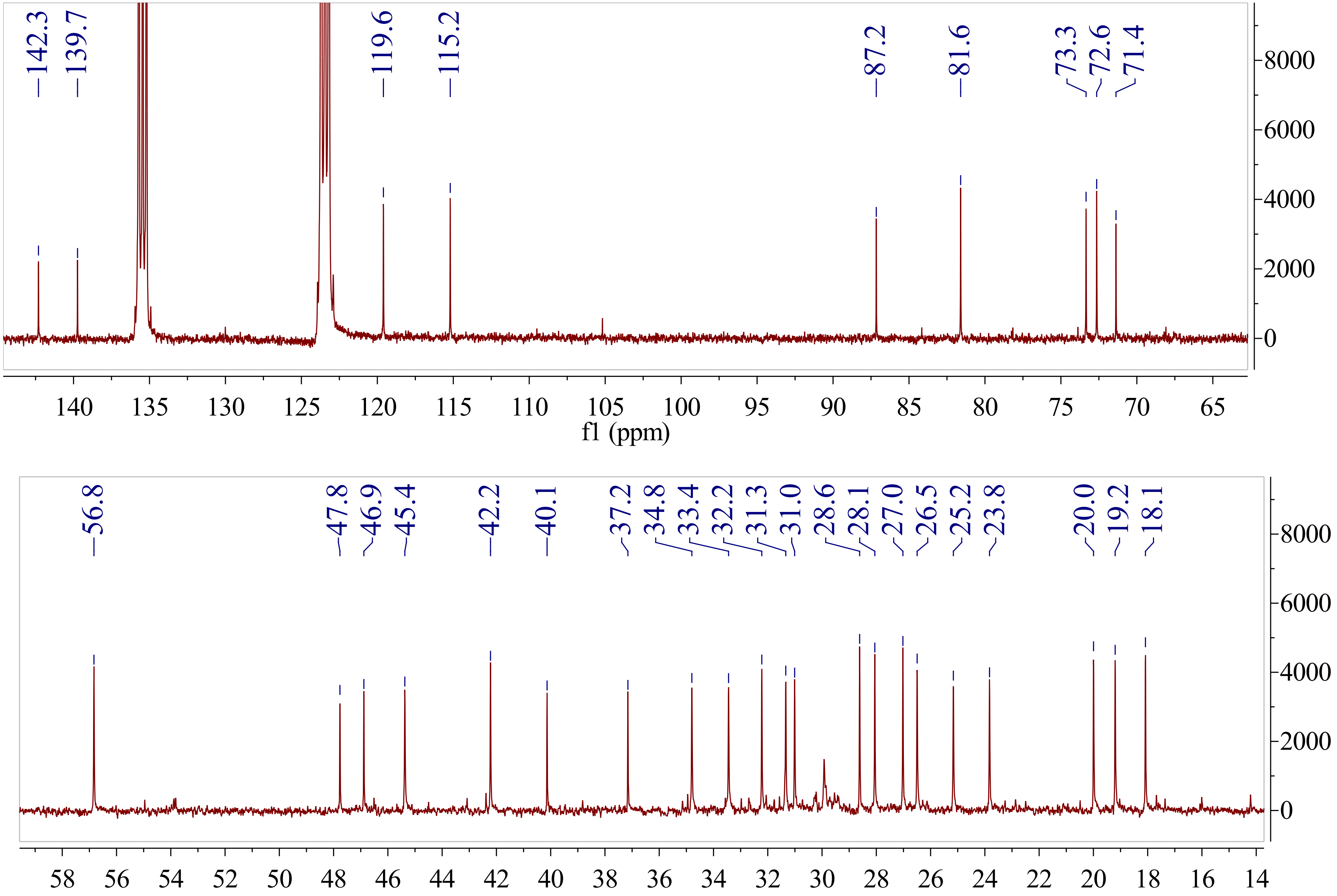

Figure S16 Amplificatory ^{13}C NMR spectrum of compound 14

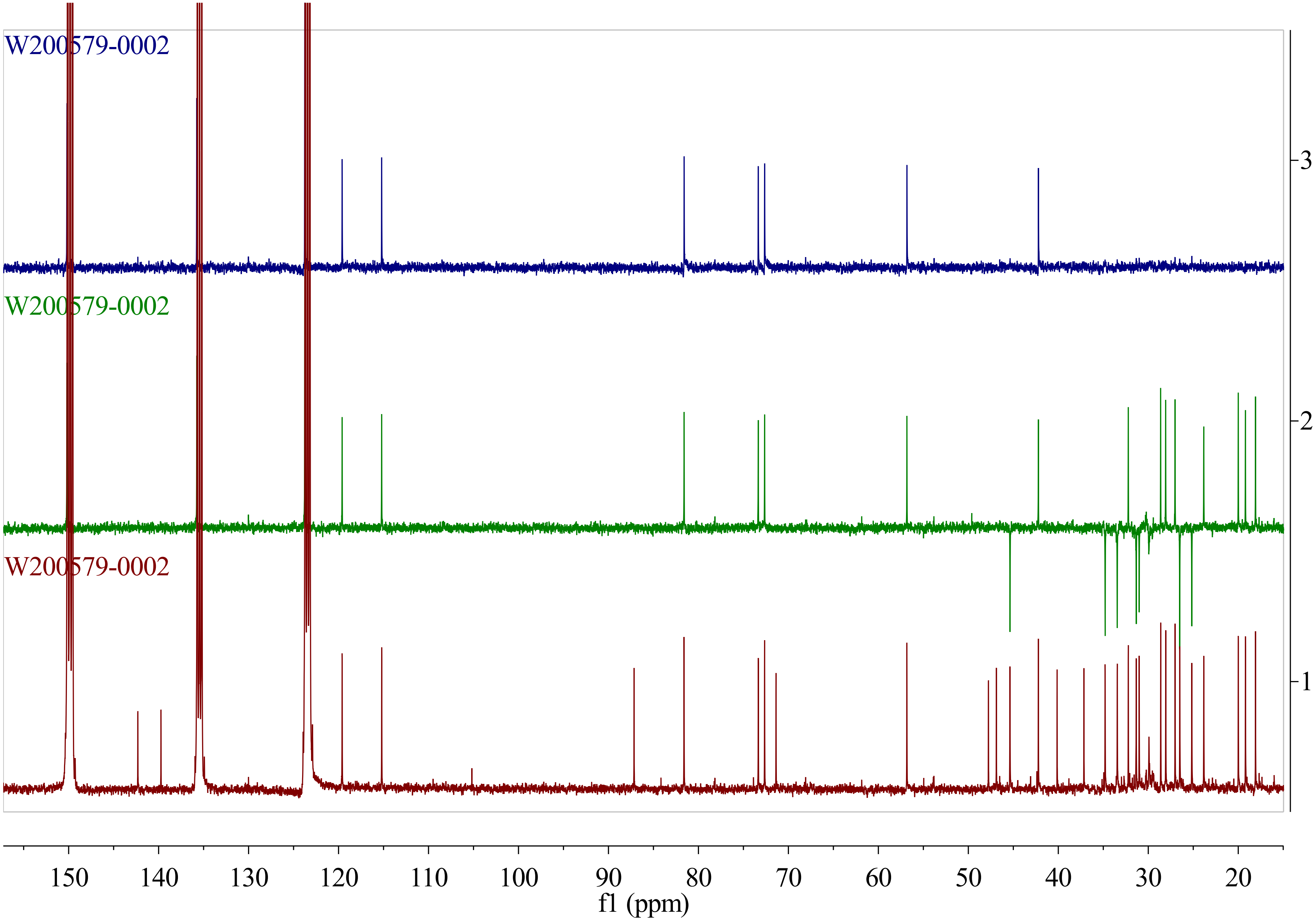

Figure S17 DEPT spectrum of compound 14

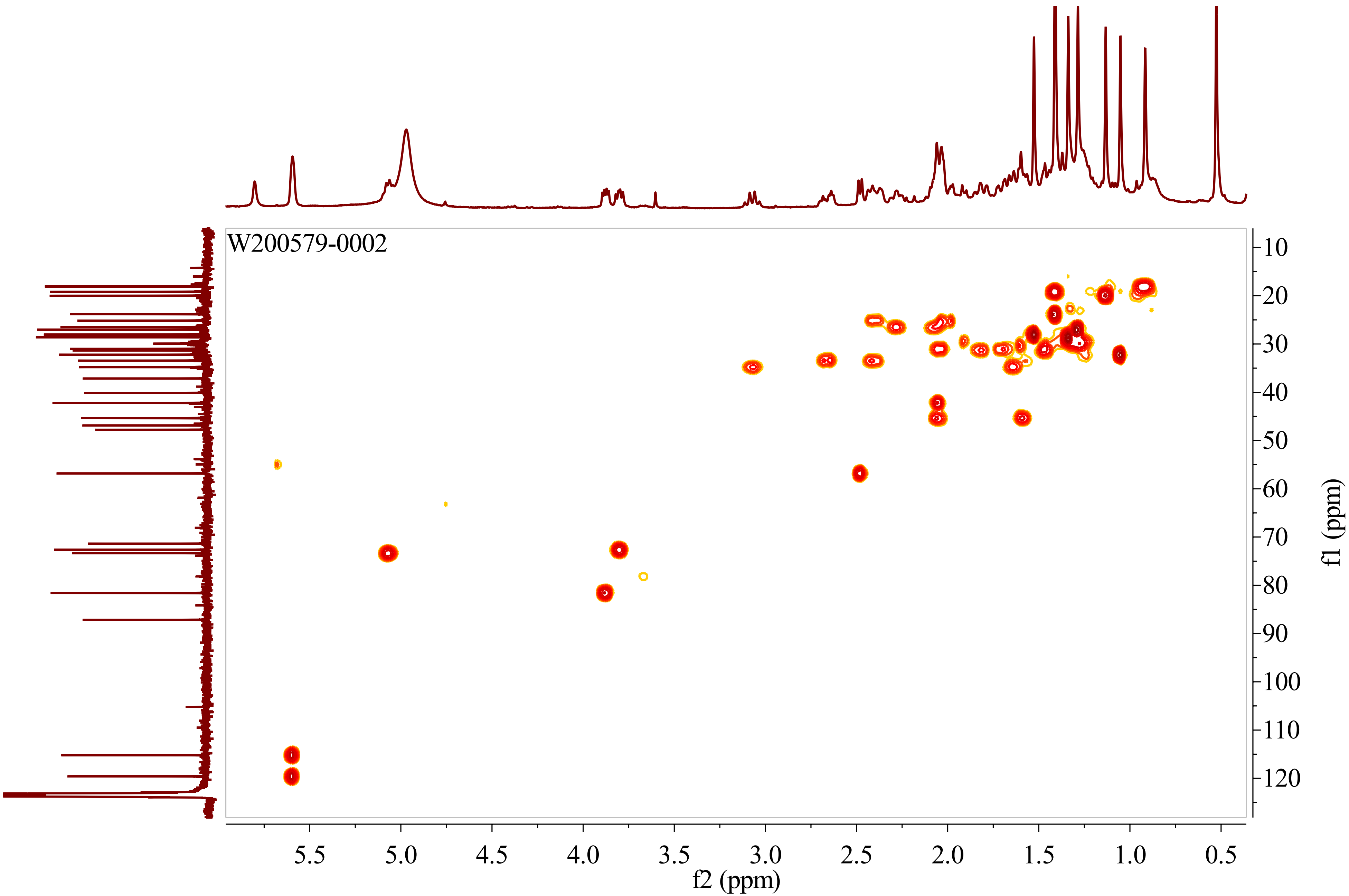

Figure S18 HMQC spectrum of compound 14

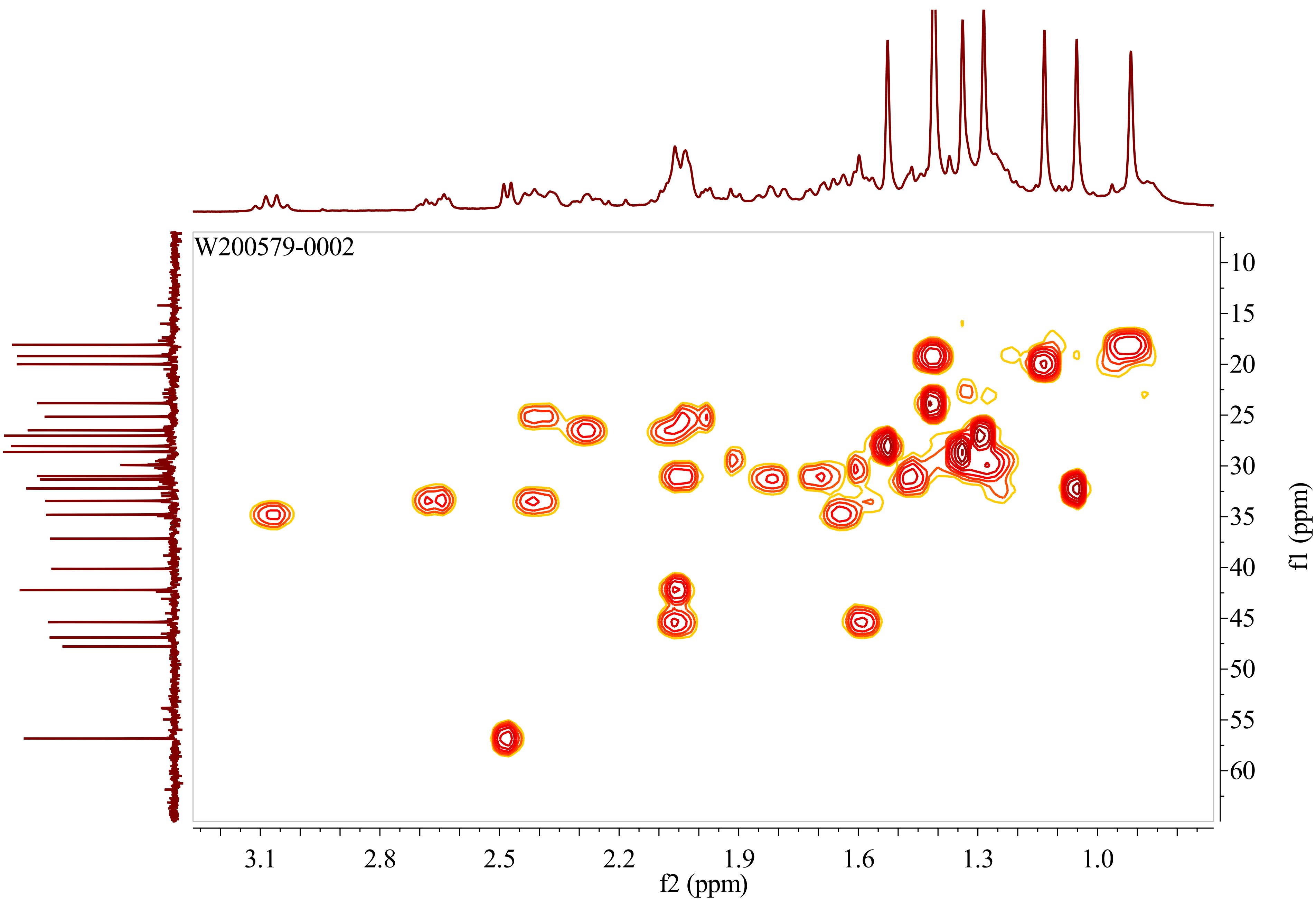

Figure S19 Amplificatory HMQC spectrum of compound 14

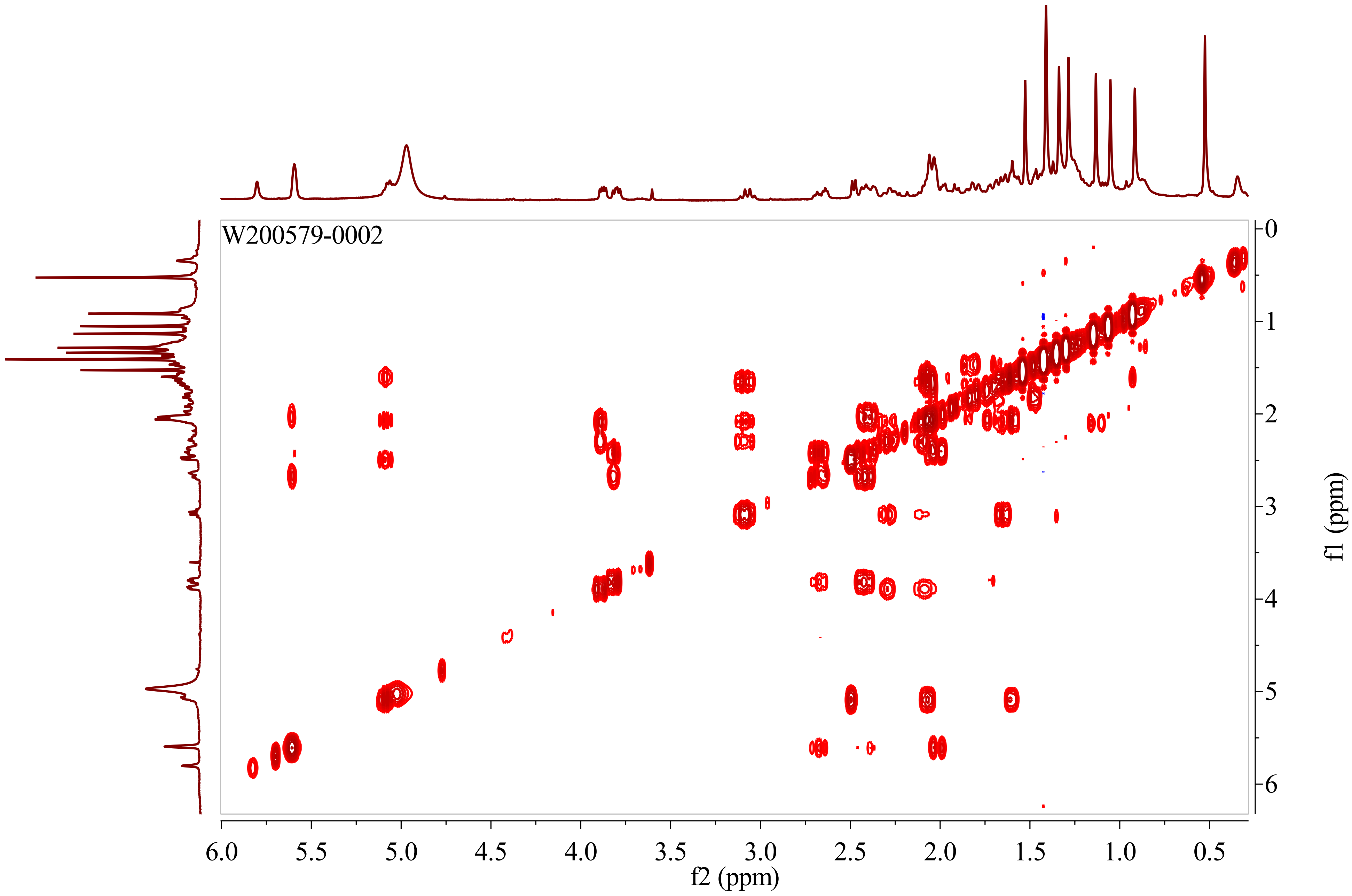

Figure S20 ^{1}H-^{1}H COSY spectrum of compound 14

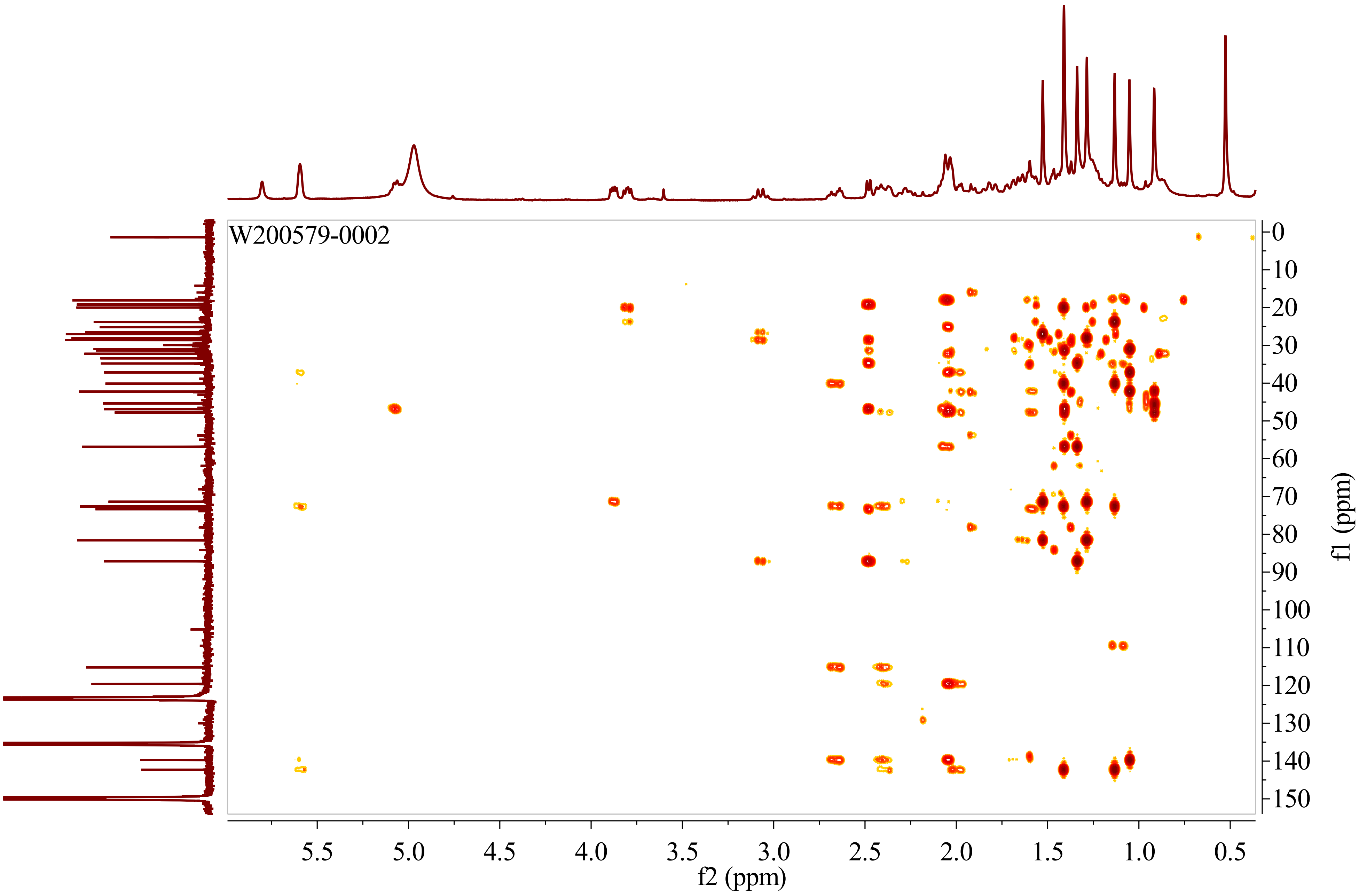

Figure S21 Key HMBC spectrum of compound 14

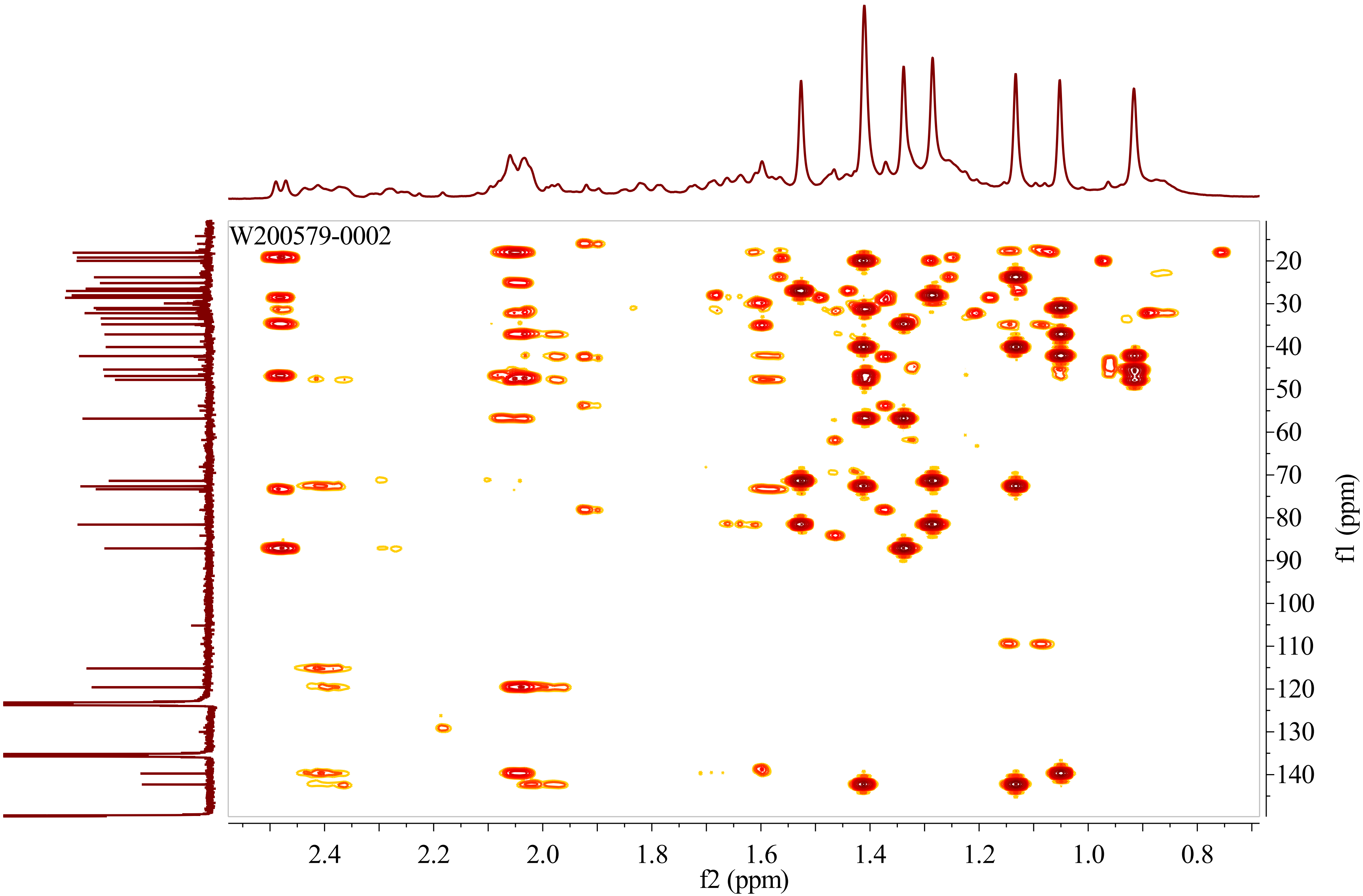

Figure S22 Amplificatory HMBC spectrum of compound 14 (A)

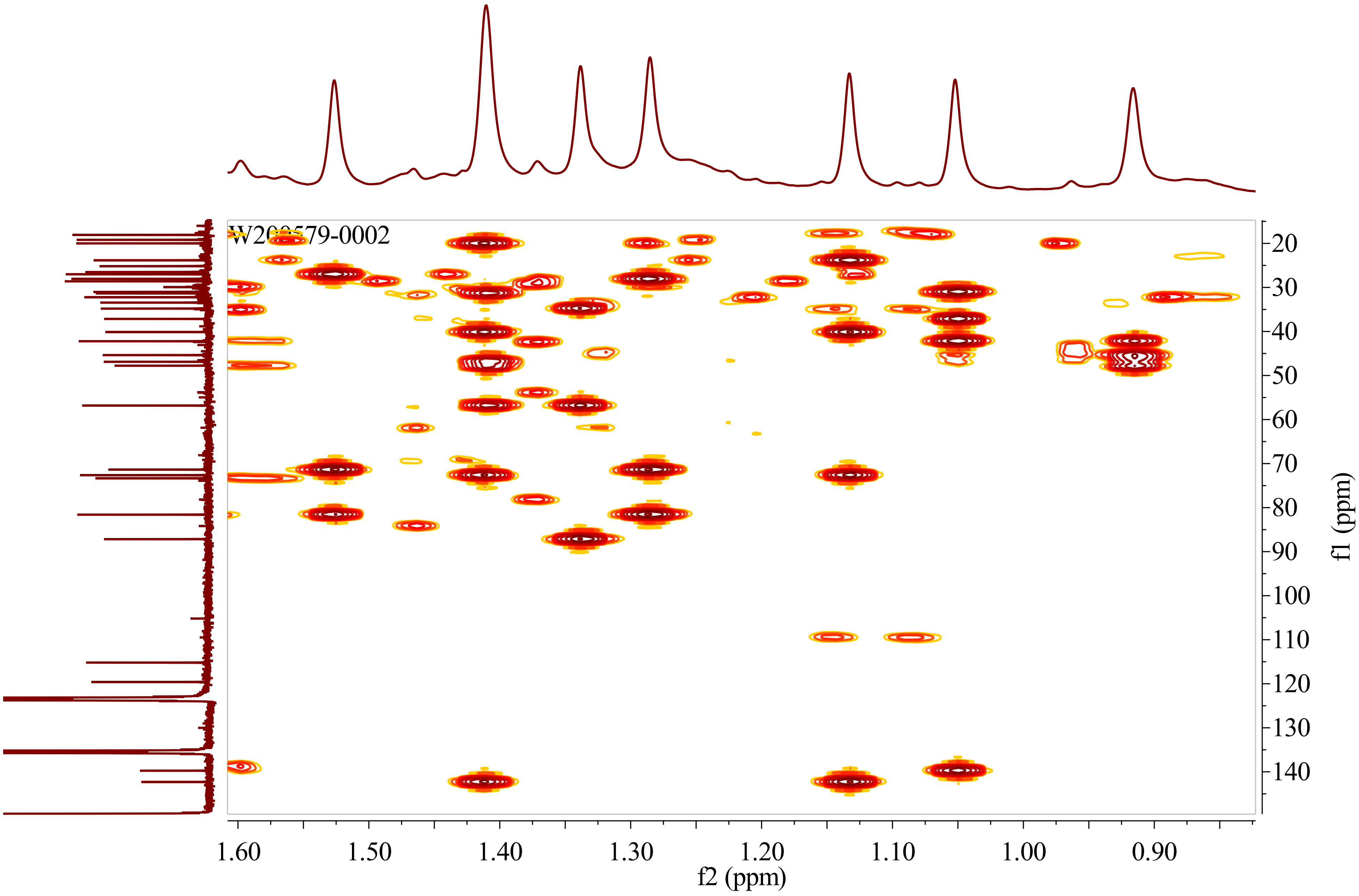

Figure S23 Amplificatory HMBC spectrum of compound 14 (B)

Table S1. ^{13}C NMR spectral data of compounds **9**, **14** and CAG.

C	9	14	CAG	C	9	14	CAG
1	35.5 (CH_2)	115.2 (CH)	31.5 (CH_2)	16	73.0 (CH)	73.3 (CH)	73.4 (CH)
2	26.5 (CH_2)	33.4 (CH_2)	32.8 (CH_2)	17	57.4 (CH)	56.8 (CH)	58.4 (CH)
3	82.7 (CH)	72.6 (CH)	78.3 (CH)	18	19.0 (CH_3)	19.2 (CH_3)	21.6 (CH_3)
4	46.1 (C)	40.1 (C)	42.4 (C)	19	35.8 (CH_3)	32.2 (CH_3)	31.0 (CH_2)
5	61.4 (CH)	142.3 (C)	54.0 (CH)	20	87.1 (C)	87.2 (C)	87.2 (C)
6	65.9 (CH)	119.6 (CH)	68.3 (CH)	21	28.6 (CH_3)	28.6 (CH_3)	28.2 (CH_3)
7	34.6 (CH_2)	25.2 (CH_2)	38.8 (CH_2)	22	34.8 (CH_2)	34.8 (CH_2)	34.9 (CH_2)
8	46.6 (CH)	42.2 (CH)	47.3 (CH)	23	26.5 (CH_2)	26.5 (CH_2)	26.4 (CH_2)
9	35.5 (C)	37.2 (C)	21.0 (C)	24	81.6 (CH)	81.6 (CH)	81.7 (CH)
10	93.2 (C)	139.7 (C)	29.9 (C)	25	71.4 (C)	71.4 (C)	71.2 (C)
11	32.8 (CH_2)	31.0 (CH_2)	26.3 (CH_2)	26	27.0 (CH_3)	27.0 (CH_3)	27.1 (CH_3)
12	32.4 (CH_2)	31.3 (CH_2)	33.4 (CH_2)	27	28.1 (CH_3)	28.1 (CH_3)	28.6 (CH_3)
13	45.6 (C)	46.9 (C)	45.0 (C)	28	23.1 (CH_3)	23.8 (CH_3)	29.4 (CH_3)
14	45.7 (C)	47.8 (C)	46.2 (C)	29	26.1 (CH_3)	20.0 (CH_3)	16.1 (CH_3)
15	46.0 (CH_2)	45.4 (CH_2)	46.8 (CH_2)	30	19.8 (CH_3)	18.1 (CH_3)	20.2 (CH_3)

Table S2 Anti-inflammatory of compound **9** activities against LPS-induced lymphocyte cells

NO.		Cell survival (%)
DMSO Control		100.00
LPS Control		77.11
Compound **9**		
	10 nM	75.18
	100 nM	85.03
	1 μM	89.57

Table S3 Anti-inflammatory of compound **14** activities against LPS-induced lymphocyte cells

NO.		Cell survival (%)
DMSO Control		100.00
LPS Control		61.35
Compound **14**		
	10 nM	67.54
	100 nM	79.46
	1 μM	89.15

Table S4 Anti-inflammatory of compound **9** and **14** activities against CSE-induced MLE-12 cells

NO.		CD69+Cell survival (%)
DMSO Control		13.15
CSE Control		59.03
Compound **9**		
	3 μM	57.96
	10 μM	56.17
	30 μM	49.61
Compound **14**		
	3 μM	60.14
	10 μM	59.03
	30 μM	50.40

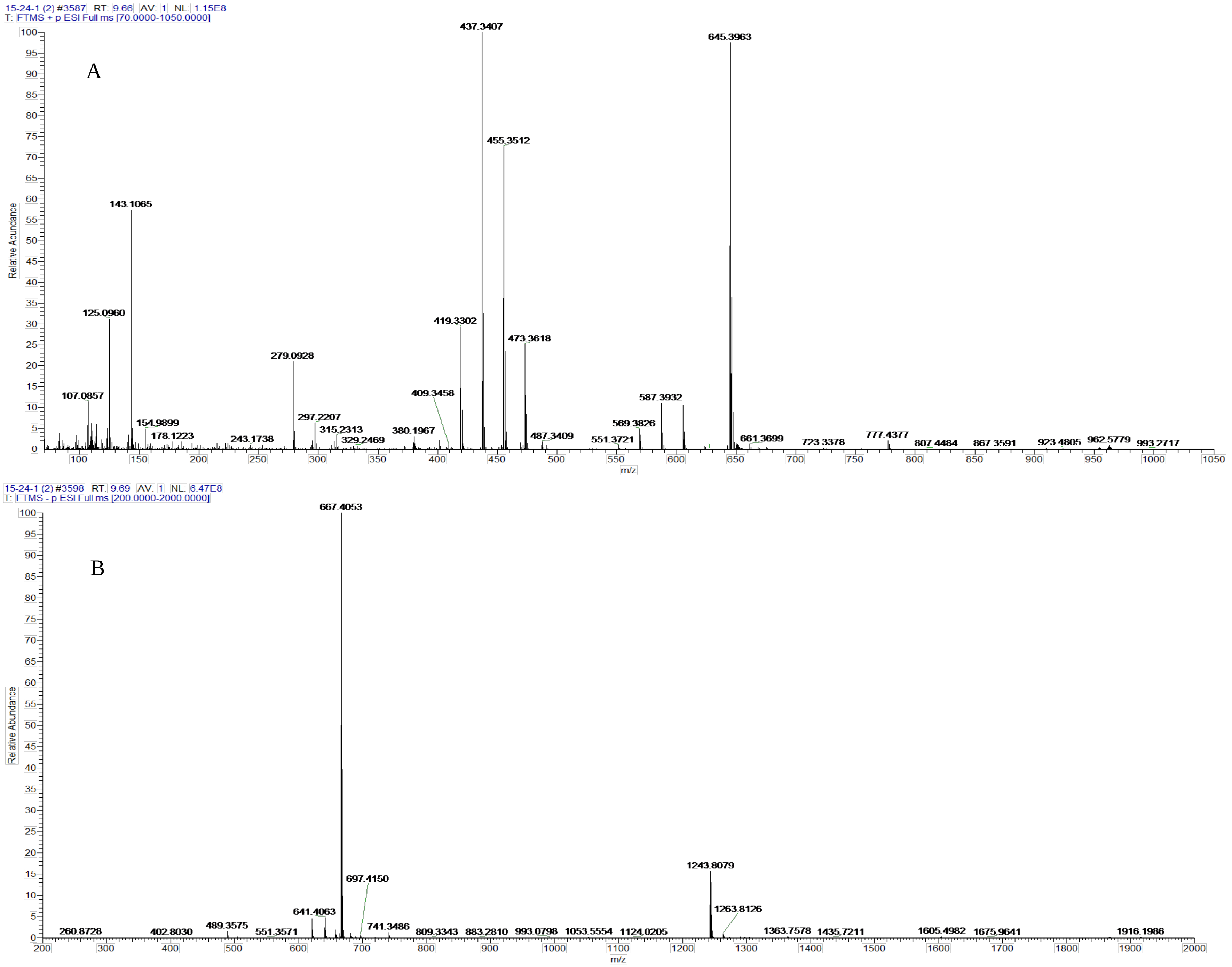

Figure S24 Positive ions (A) and negative ions (B) ESI-MS/MS spectra of compound **1**

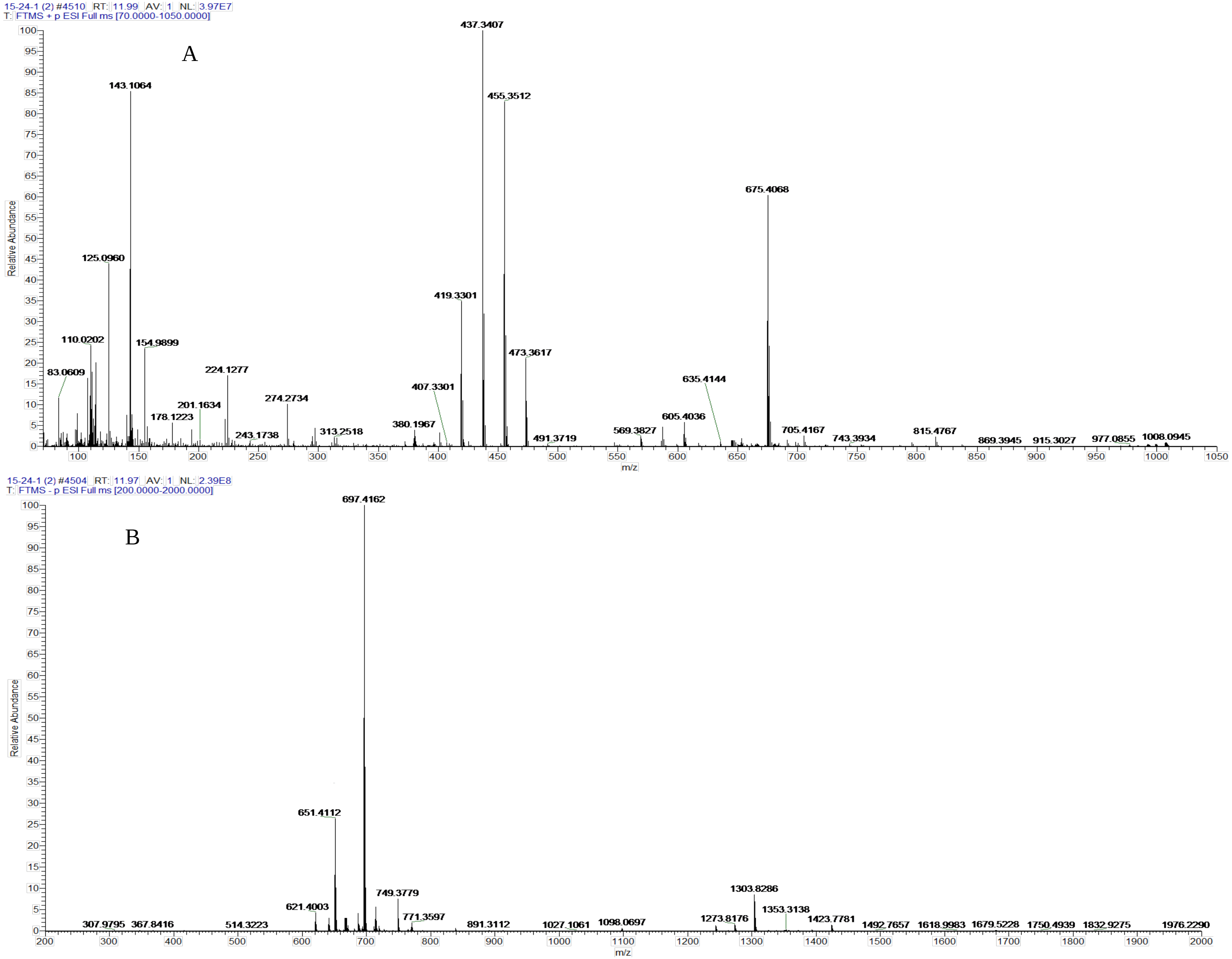

Figure S25 Positive ions (A) and negative ions(B) ESI-MS/MS spectra of compound **2**

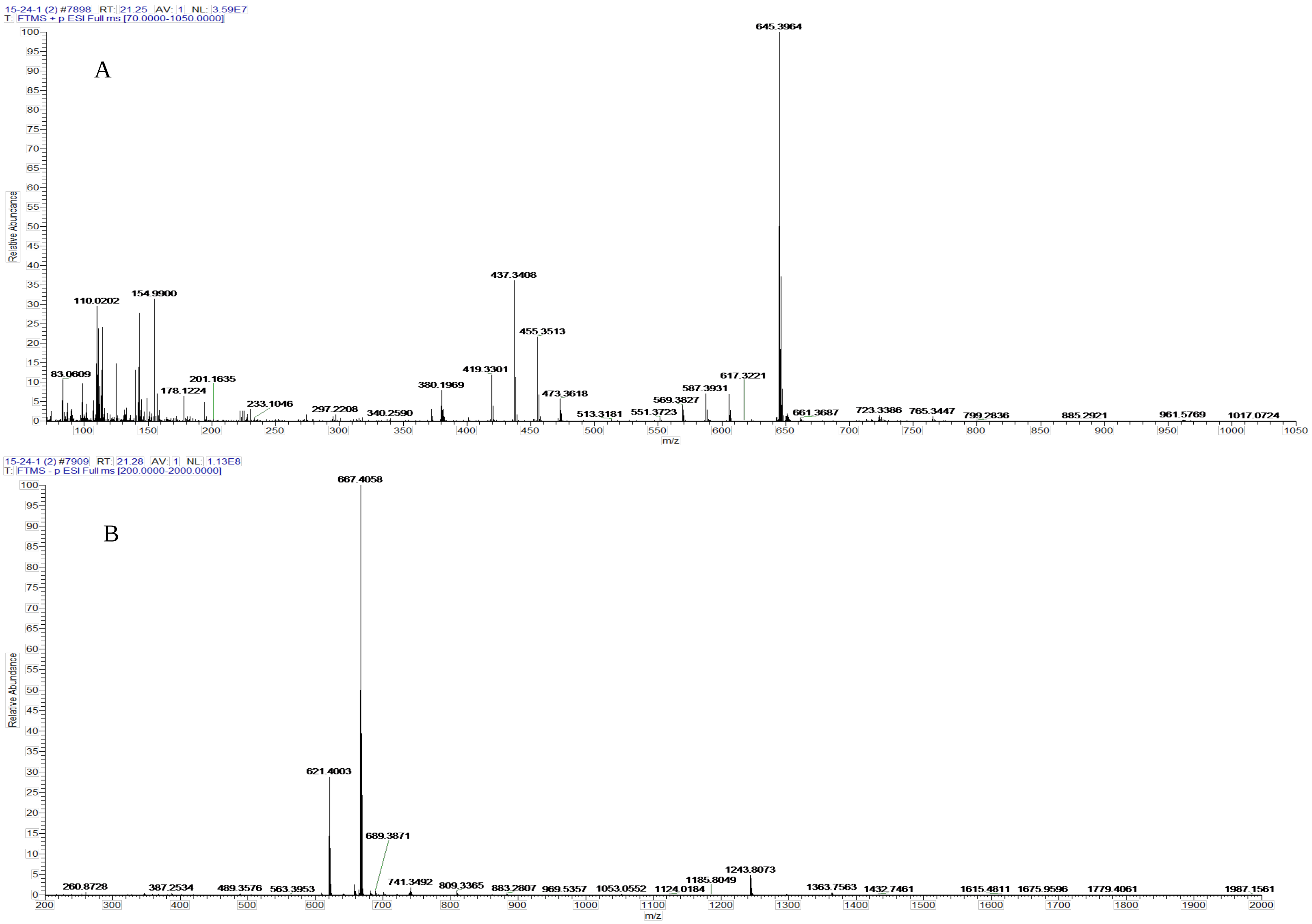

Figure S26 Positive ions (A) and negative ions (B) ESI-MS/MS spectra of compound **3**

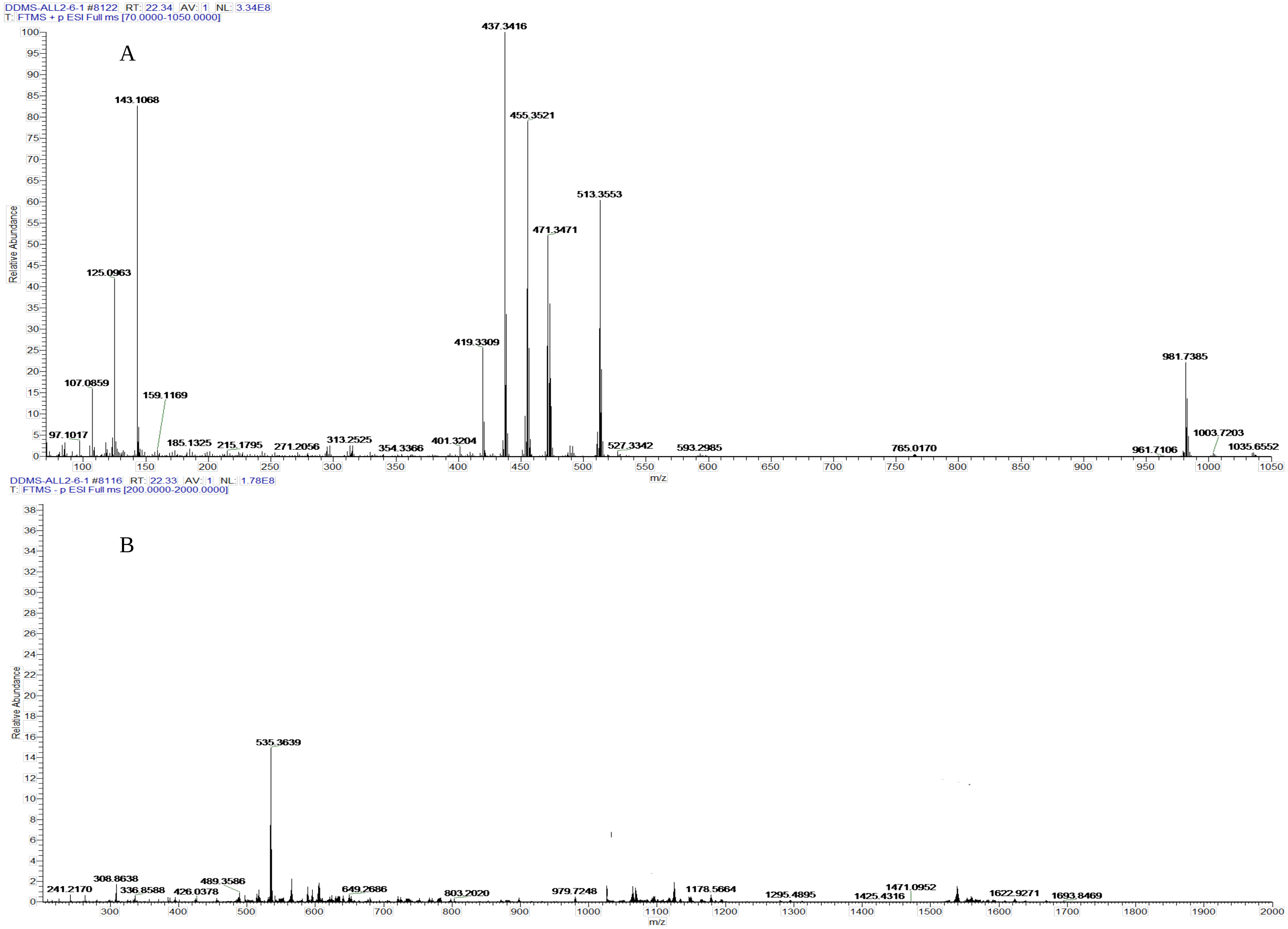

Figure S27 Positive ions (A) and negative ions (B) ESI-MS/MS spectra of compound **4**

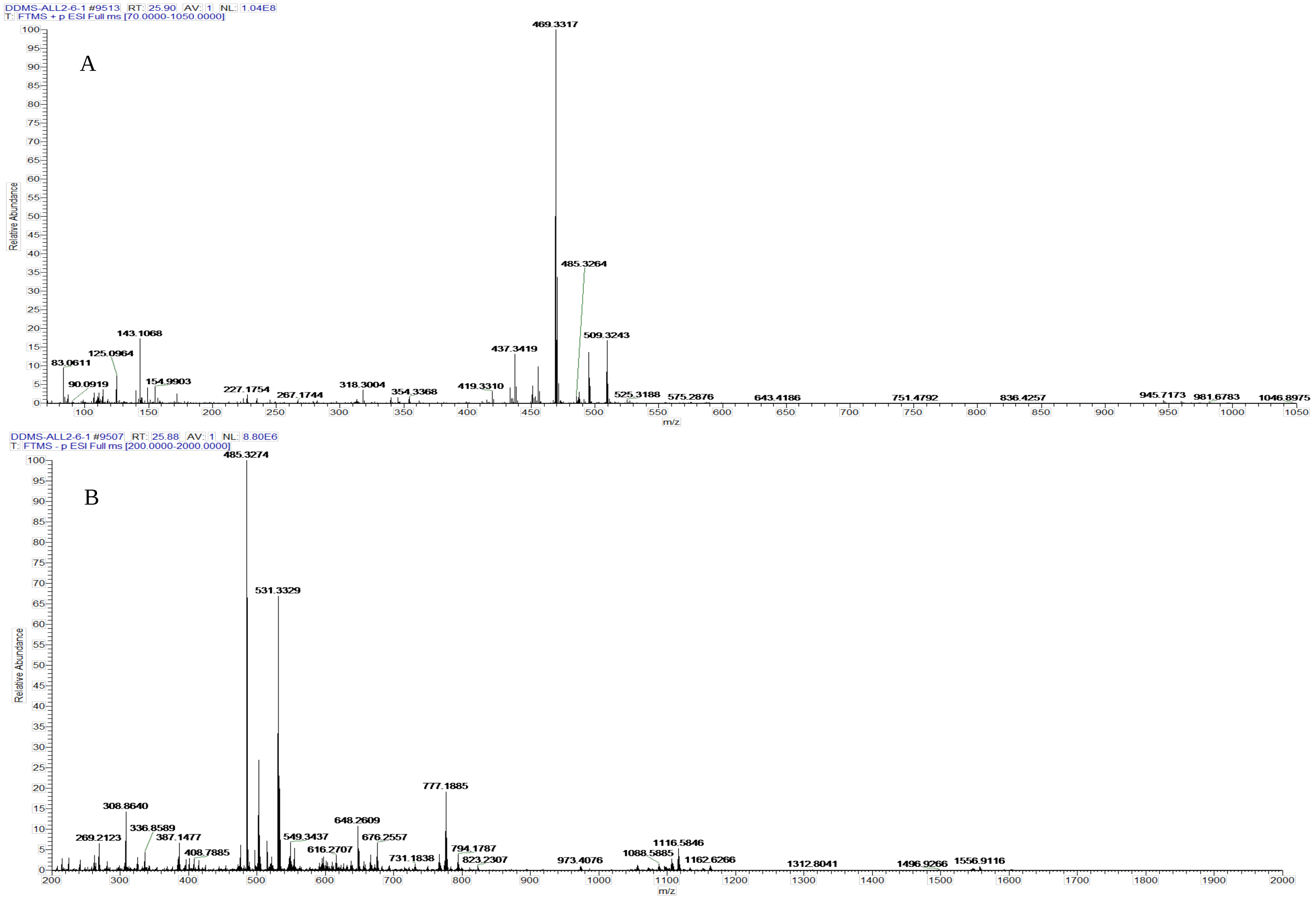

Figure S28 Positive ions (A) and negative ions (B) ESI-MS/MS spectra of compound **5**

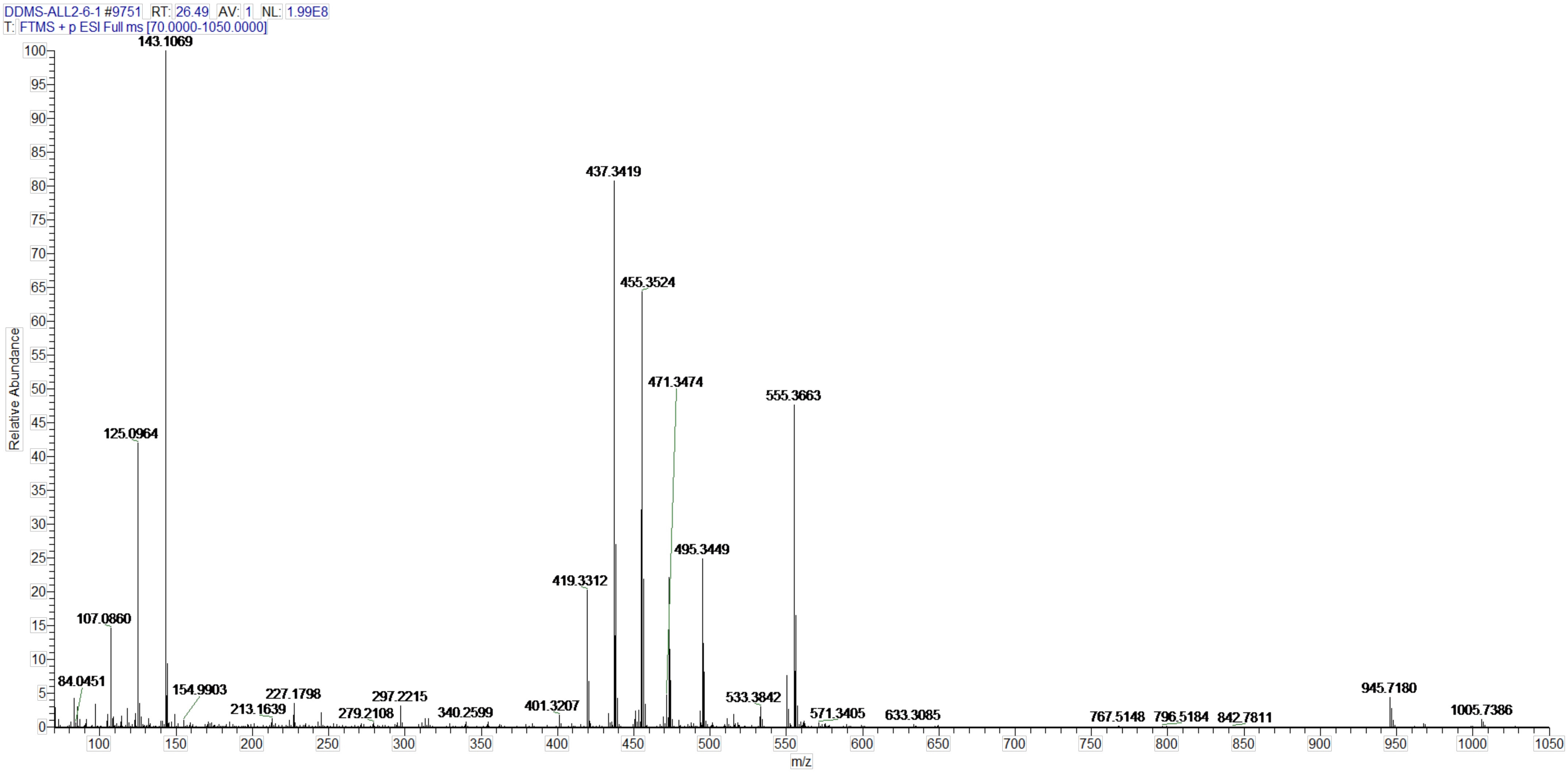

Figure S29 Positive ions ESI-MS/MS spectra of compound **6**

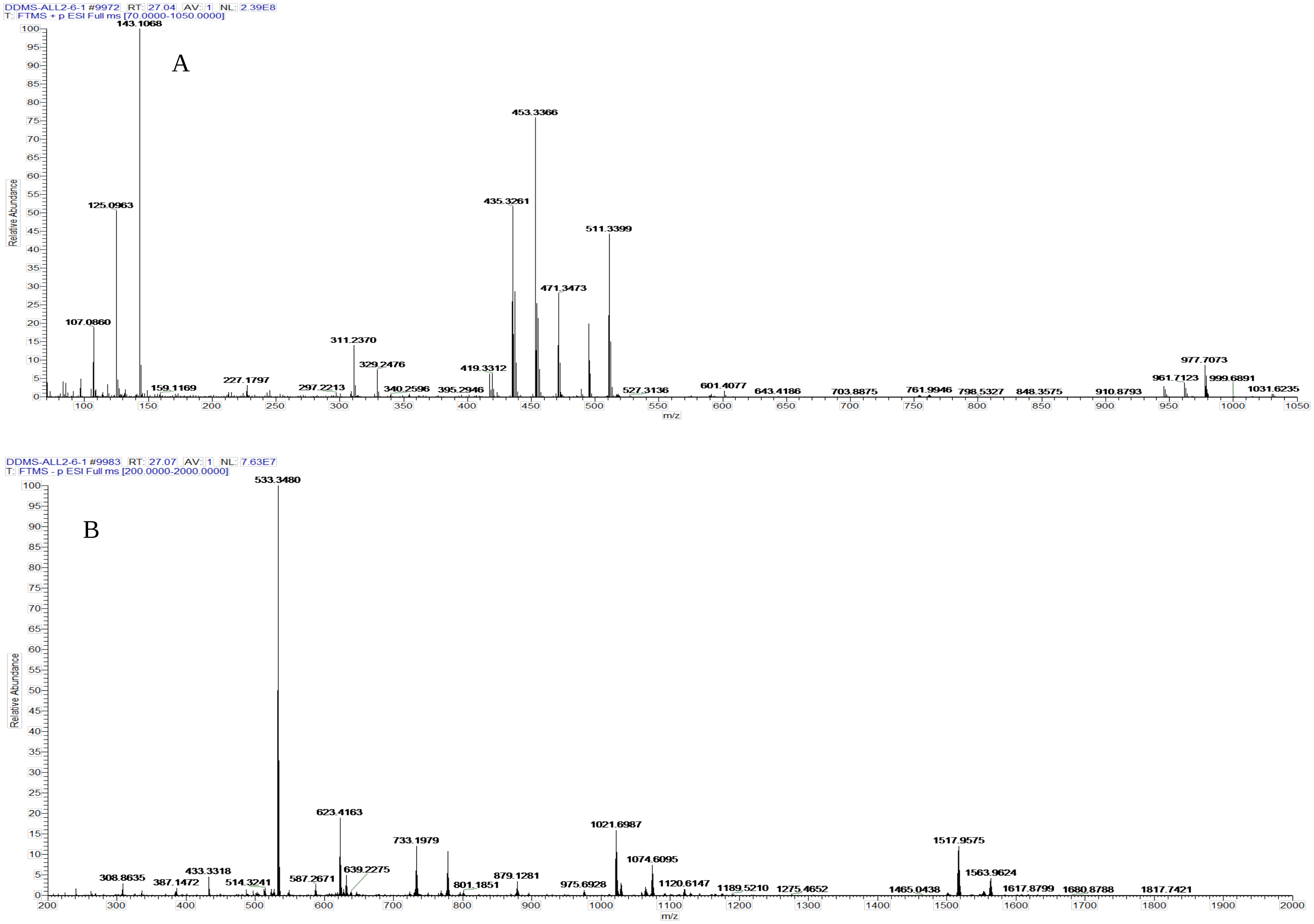

Figure S30 Positive ions (A) and negative ions (B) ESI-MS/MS spectra of compound **7**

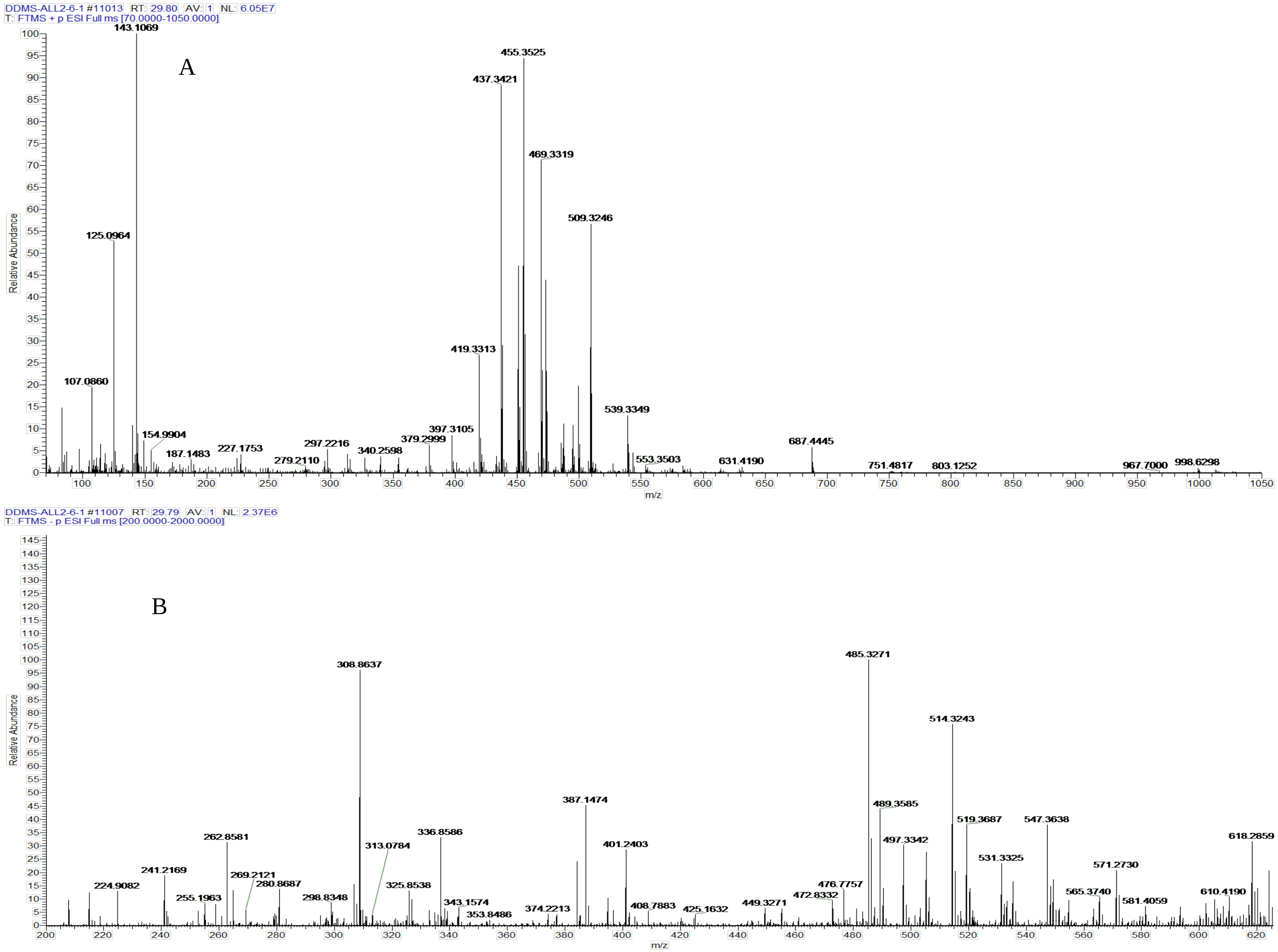

Figure S31 Positive ions (A) and negative ions (B) ESI-MS/MS spectra of compound **8**

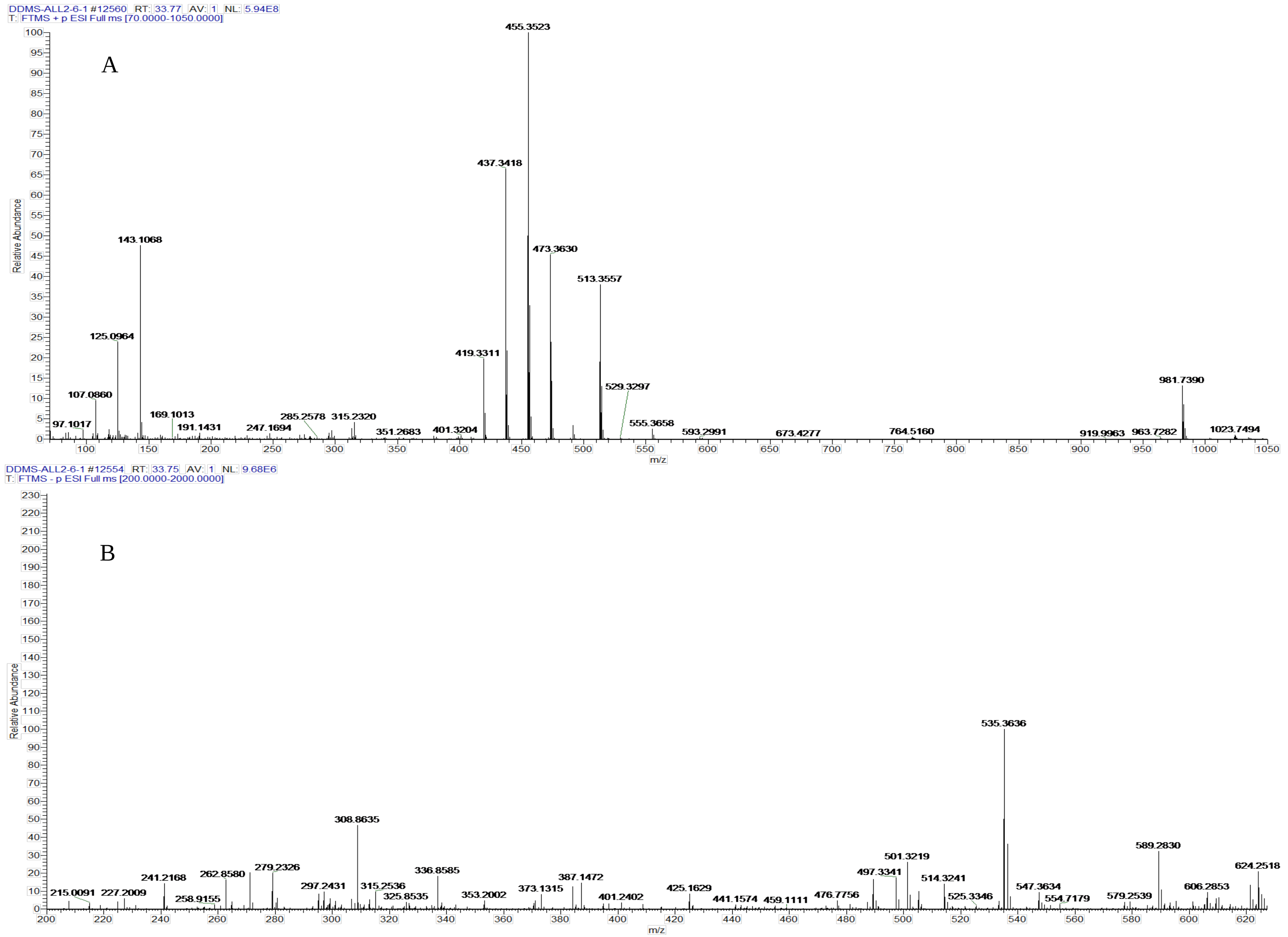

Figure S32 Positive ions (A) and negative ions (B) ESI-MS/MS spectra of compound **9**

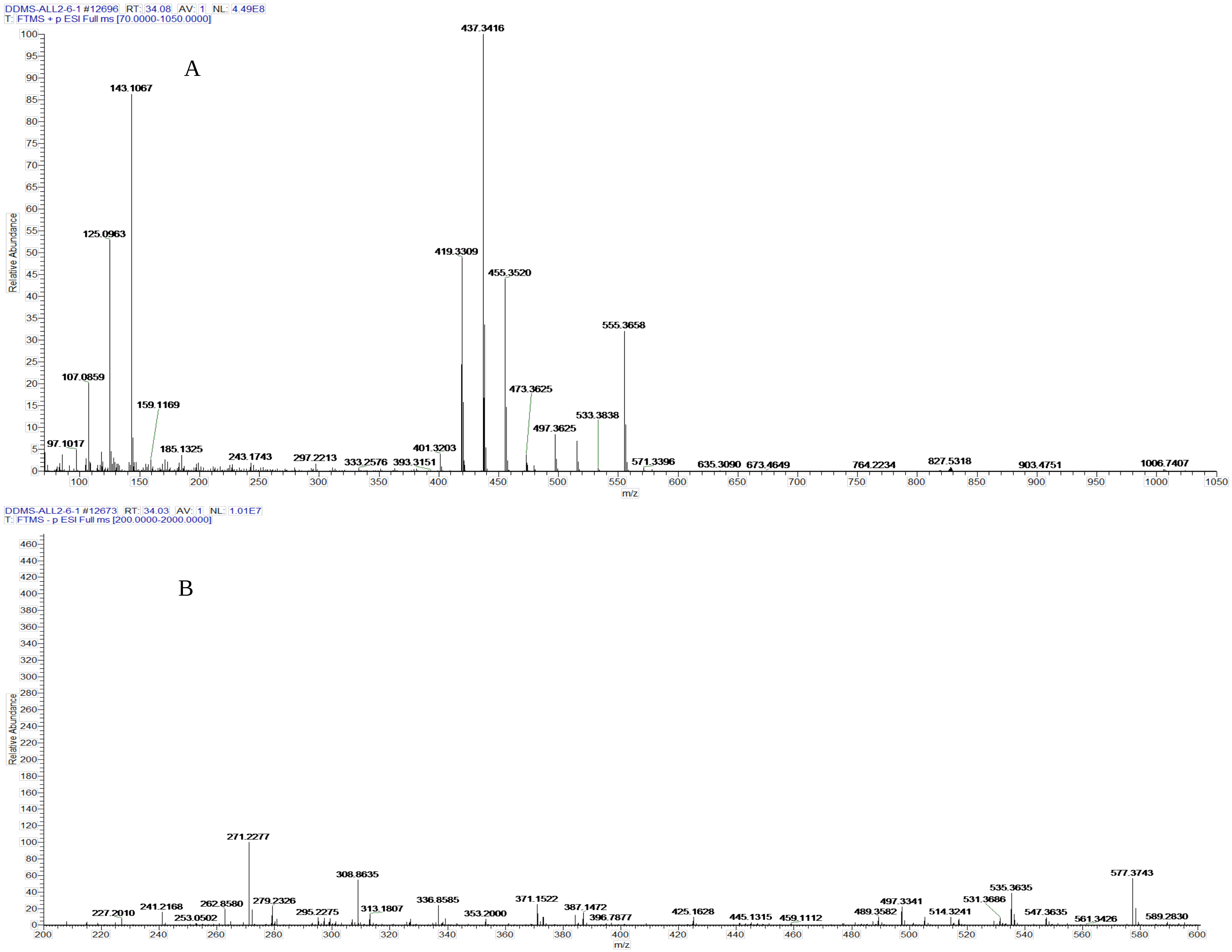

Figure S33 Positive ions (A) and negative ions (B) ESI-MS/MS spectra of compound **10**

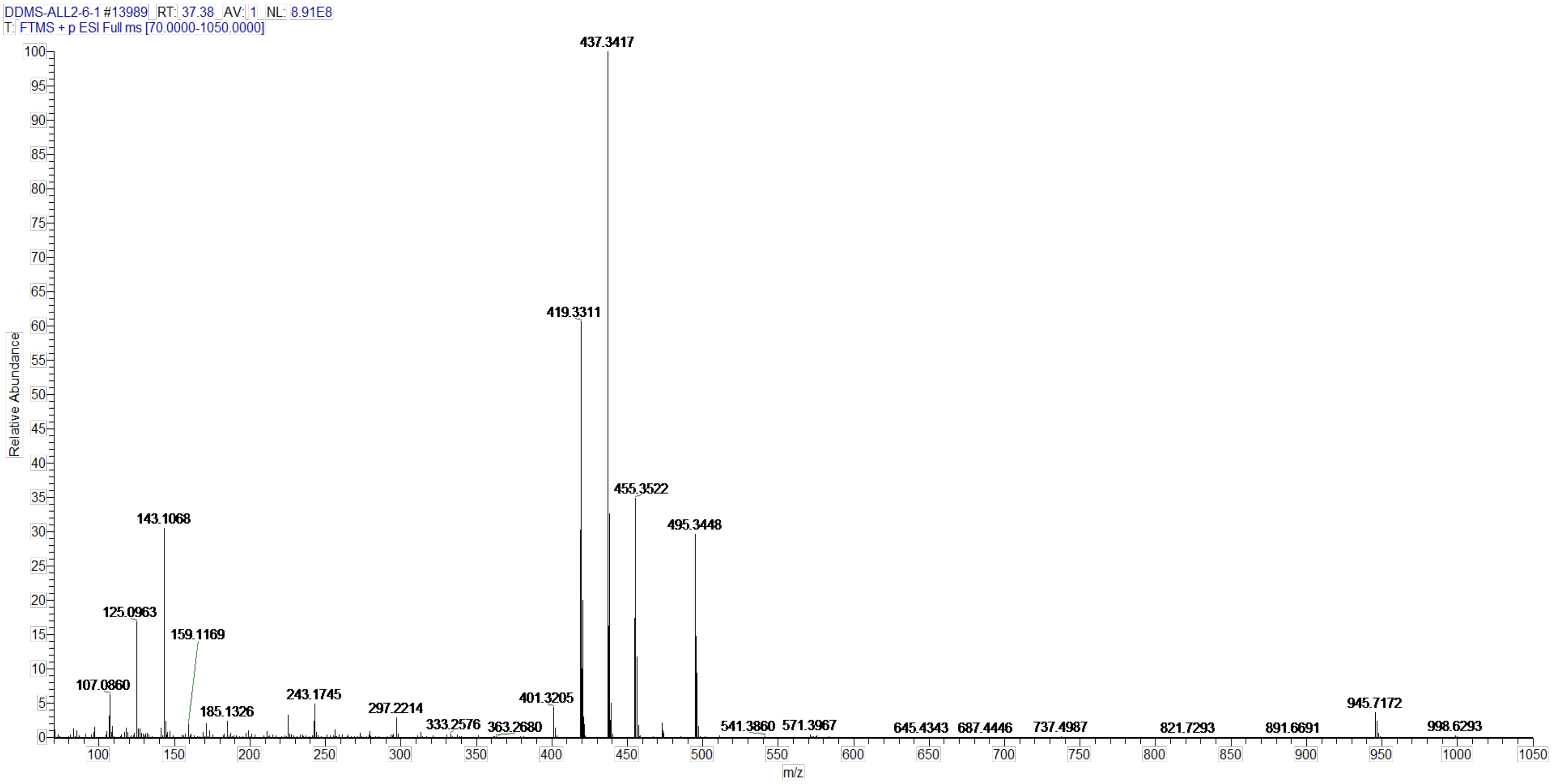

Figure S34 Positive ions ESI-MS/MS spectra of compound **11**

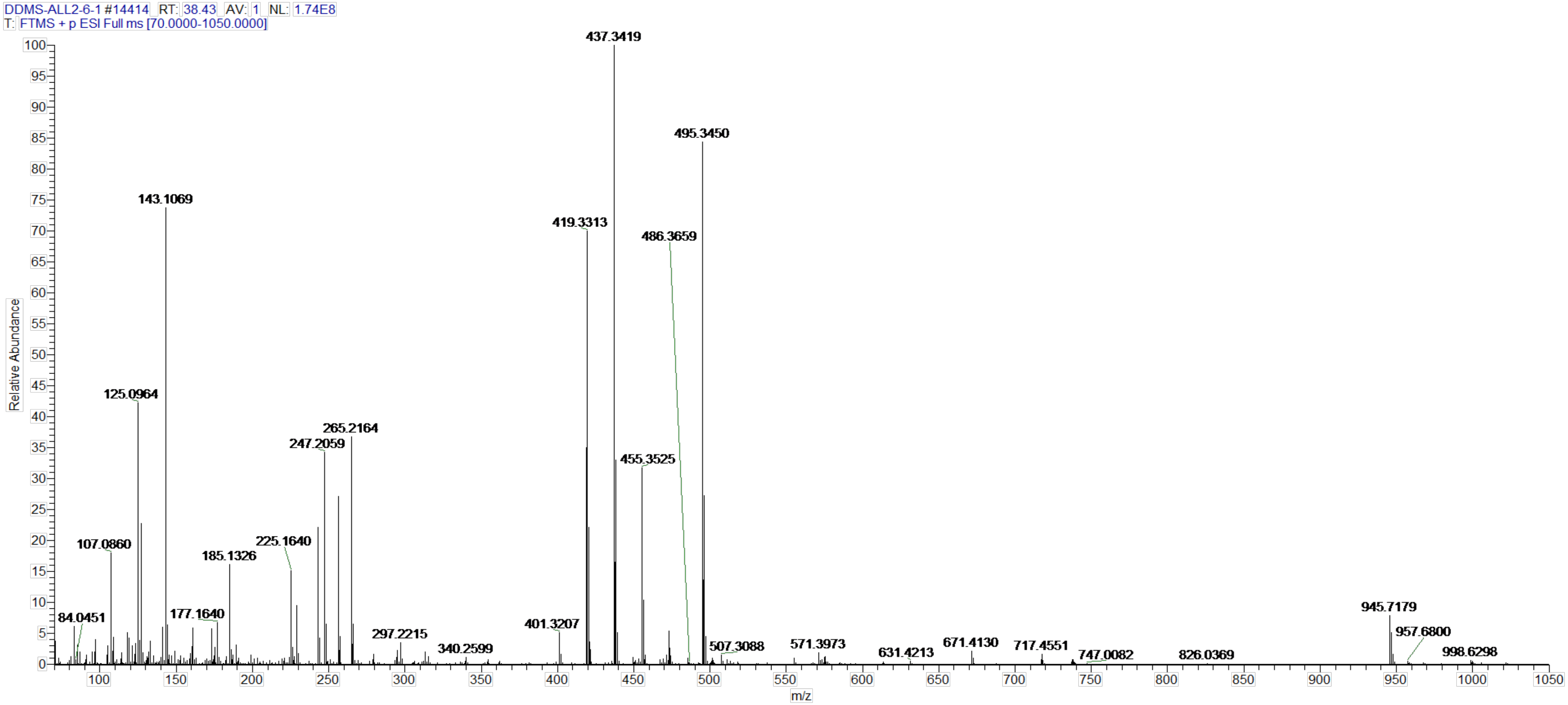

Figure S35 Positive ions ESI-MS/MS spectra of compound **12**

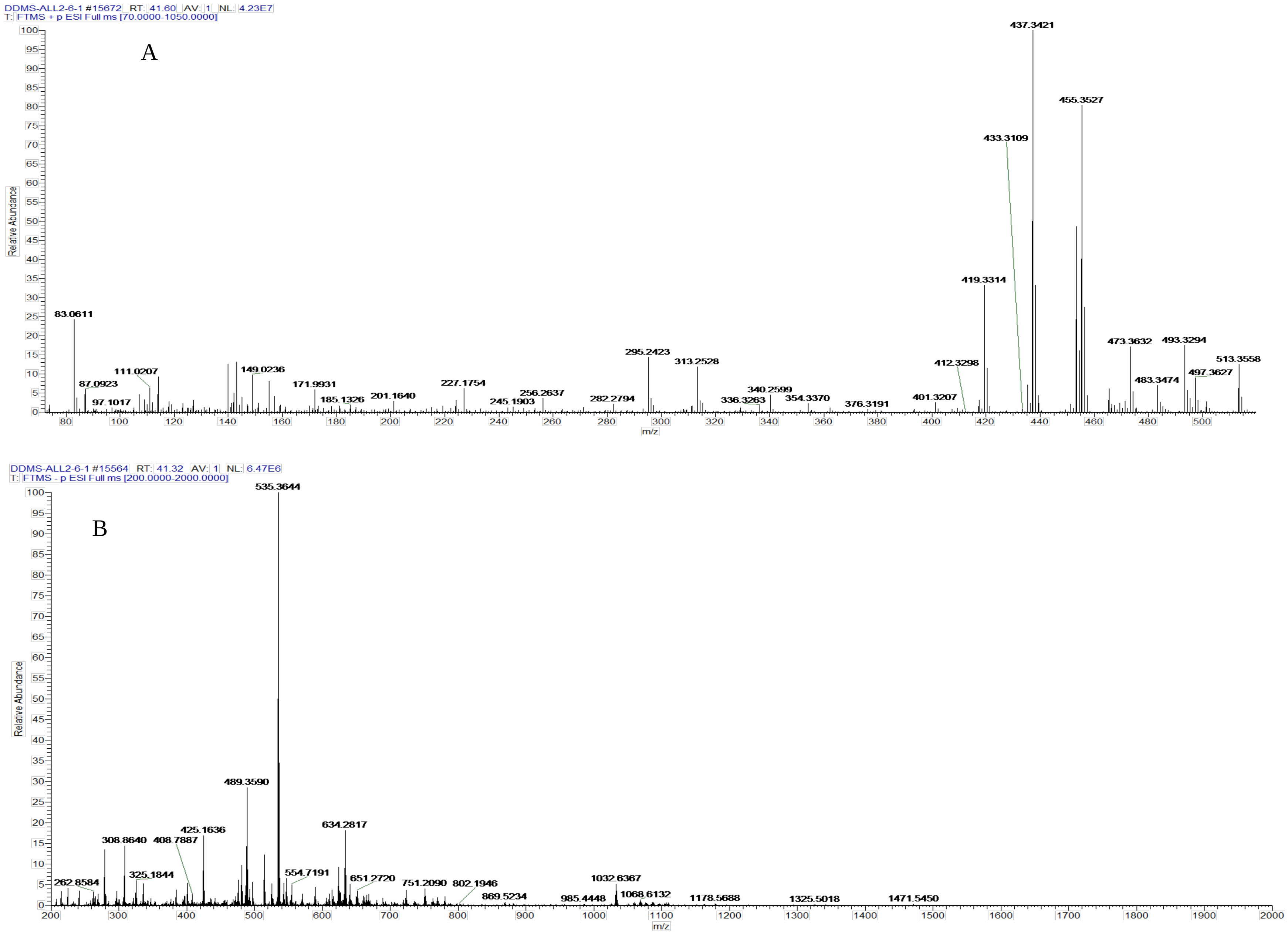

Figure S36 Positive ions (A) and negative ions (B) ESI-MS/MS spectra of compound **13**

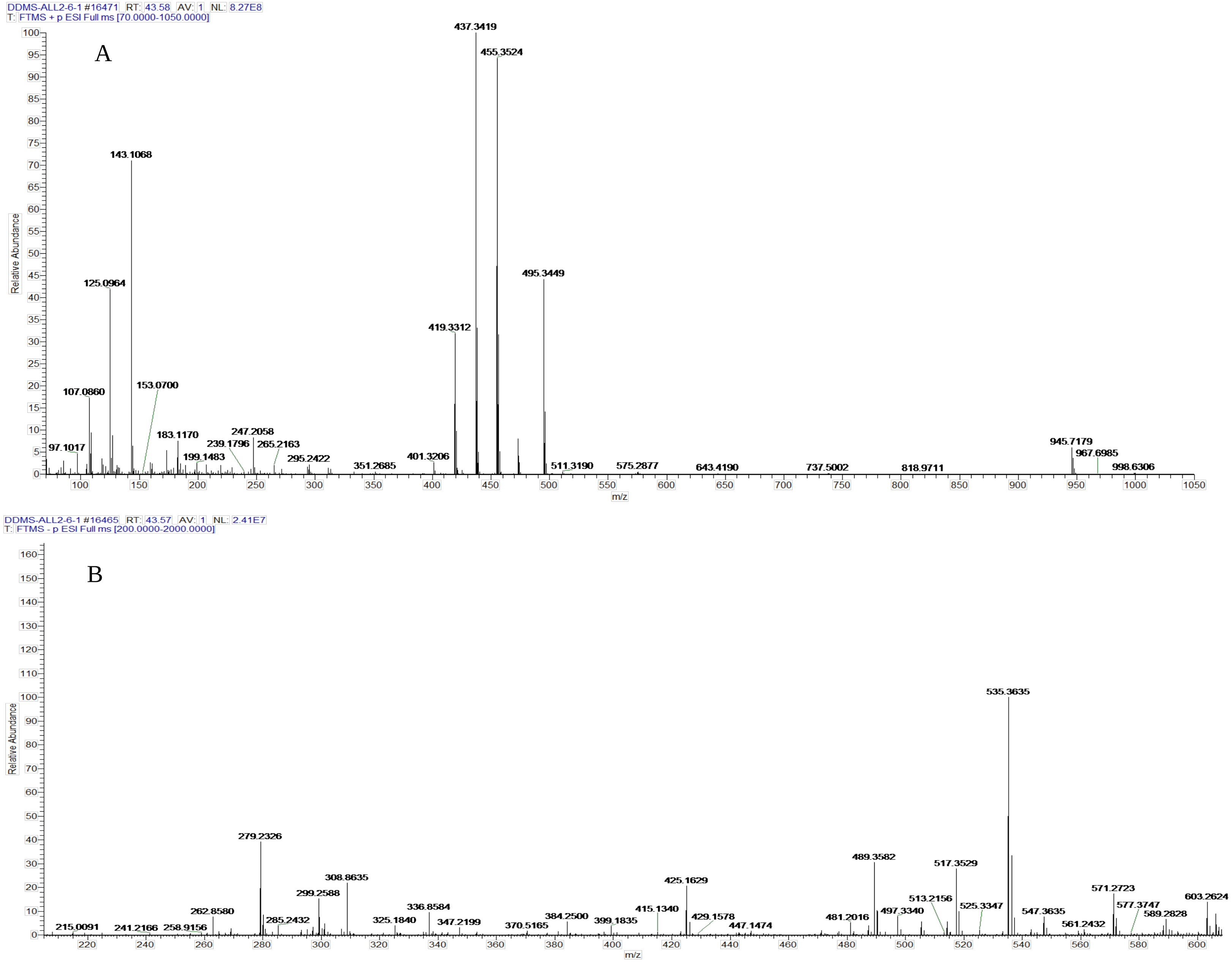

Figure S37 Positive ions (A) and negative ions (B) ESI-MS/MS spectra of compound **14**

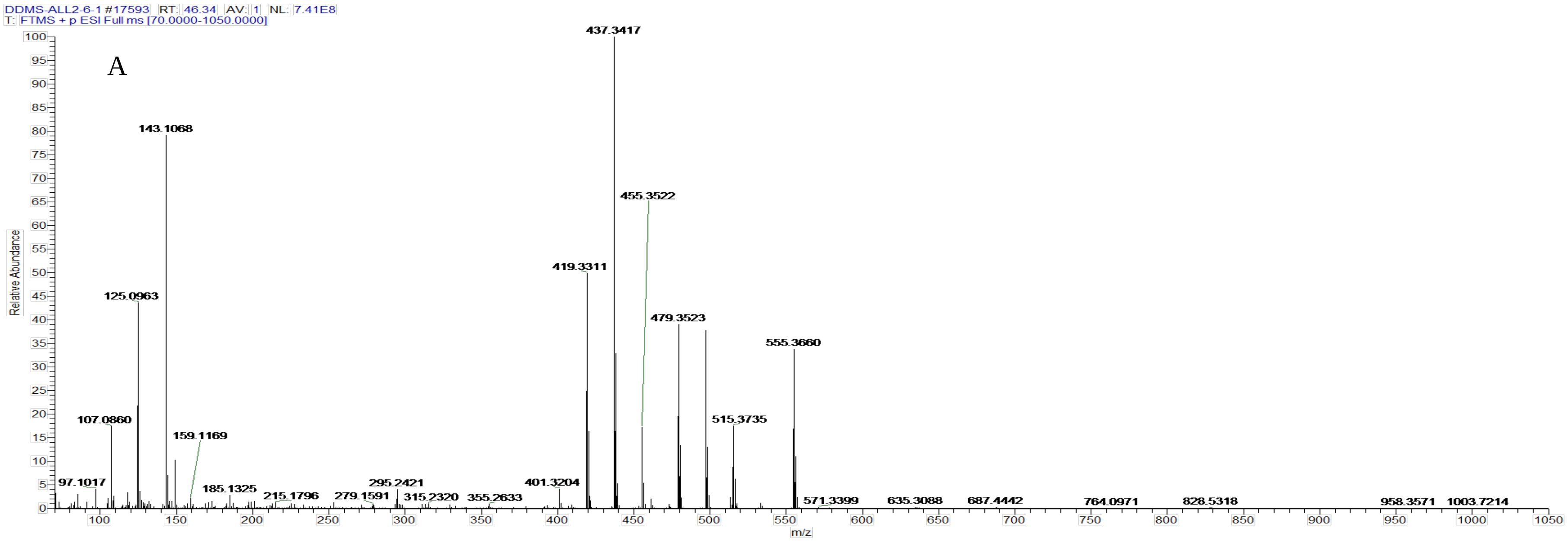

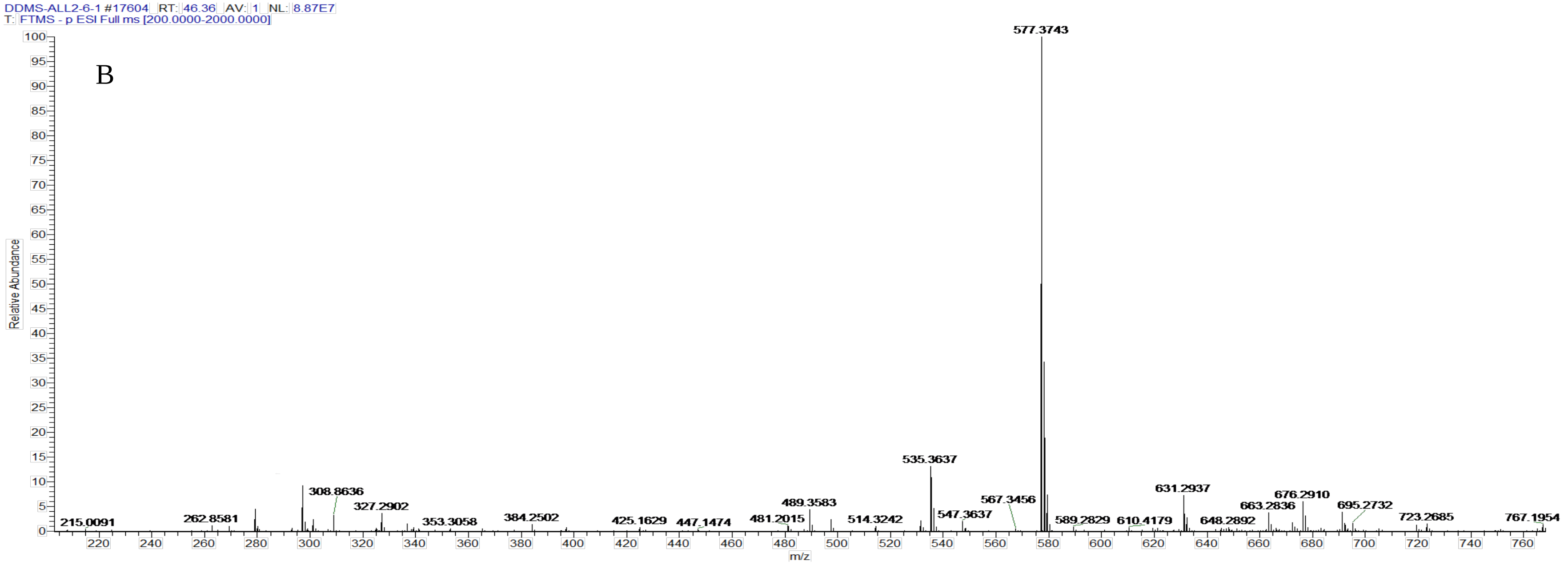

Figure S38 Positive ions (A) and negative ions (B) ESI-MS/MS spectra of compound **15**
